# *Phocaeicola coprophilus*‐Derived 6‐Methyluracil Attenuates Radiation‐Induced Intestinal Fibrosis by Suppressing the IDO1‐Kynurenine‐AHR Axis

**DOI:** 10.1002/advs.202518502

**Published:** 2026-01-20

**Authors:** Jiaxin Zhang, Zhen Wang, Shuang Li, Chao Luo, Han Li, Shengjie Ma, Pai Wang, Heshi Liu, Lijun Sun, Yue Yin, Weizhen Zhang, Quan Wang

**Affiliations:** ^1^ Department of Gastrocolorectal Surgery General Surgery Center The First Hospital of Jilin University Changchun China; ^2^ Department of Physiology and Pathophysiology School of Basic Medical Sciences and State Key Laboratory of Vascular Homeostasis and Remodeling Peking University Beijing P. R. China; ^3^ Department of Pharmacology School of Basic Medical Sciences and State Key Laboratory of Vascular Homeostasis and Remodeling Peking University Beijing P. R. China

**Keywords:** gut microbiota, IDO1, kynurenine pathway, radiation‐induced intestinal fibrosis

## Abstract

Therapeutic options for radiation‐induced intestinal fibrosis (RIF) remain limited. This study reveals that intestinal kynurenine (Kyn) is persistently elevated after radiation and correlates with fibrosis severity in both murine models and human rectal cancer samples. Exogenous Kyn exacerbated RIF, whereas inhibition of indoleamine 2,3‐dioxygenase 1 (IDO1) attenuated fibrotic progression. Mechanistically, Kyn activates the aryl hydrocarbon receptor (AHR) to promote fibroblast activation and fibrosis. Antibiotic depletion of gut microbiota abrogates radiation‐induced IDO1‐Kyn upregulation and protects against RIF. Conversely, fecal microbiota transplantation from irradiated mice recapitulates the elevated IDO1‐Kyn phenotype. Metagenomic analysis identify radiation‐induced depletion of *Phocaeicola coprophilus* (*P. coprophilus*), whose abundance inversely correlates with Kyn levels. Supplementation with live *P. coprophilus* suppresses IDO1‐Kyn signaling and ameliorates RIF. Untargeted metabolomics further show that radiation reduces 6‐methyluracil, a metabolite derived from *P. coprophilus*. Exogenous 6‐methyluracil replenishment inhibits repression of the IDO1‐Kyn axis and mitigates fibrosis. Together, these findings define a microbiota–metabolite–host pathway in which radiation depletes *P. coprophilus*, leading to loss of 6‐methyluracil and derepression of the IDO1‐Kyn‐AHR axis, thereby driving fibrogenesis. Restoration of either *P. coprophilus* or its metabolite 6‐methyluracil represents a promising therapeutic strategy against RIF.

## Graphical abstract

IR‐induced dysbiosis depletes *P. coprophilus* and its metabolite 6‐methyluracil, leading to disinhibition of the IDO1‐Kyn‐AHR axis. This results in sustained fibroblast activation and collagen deposition, driving radiation induced intestinal fibrosis.

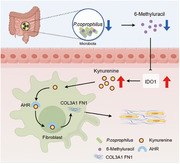

## 1 Introduction

Radiation therapy holds an indispensable position in the management of malignant tumors. Epidemiological evidence indicates that approximately 50% of cancer patients require radiotherapy during their disease course, with 25% achieving long‐term survival through this modality [1]. Despite advancements in precision techniques like 3D conformal radiotherapy and intensity‐modulated radiotherapy, radiation‐induced damage to normal organs remains inevitable due to the inherent physical properties of ionizing radiation and the radiosensitivity of adjacent tissues. During abdominal/pelvic radiotherapy, the intestine, owing to its anatomical location and high regenerative activity, is particularly vulnerable [2, 3, 4, 5]. Clinical data reveal that 60%‐80% of patients undergoing radiotherapy develop acute radiation‐induced intestinal injuries (e.g., abdominal pain, diarrhea), which are often self‐limiting. However, approximately 20% of patients progress to chronic radiation‐induced intestinal injury, characterized by irreversible complications such as intestinal obstruction and fistulae, severely compromising quality of life [1]. Radiation‐induced intestinal fibrosis (RIF) constitutes the core pathological process underlying these chronic injuries [6, 7]. Consequently, suppressing intestinal fibrosis is paramount for improving patient prognosis. Unfortunately, clinical interventions targeting the fibrotic process remain limited. Therefore, elucidating the molecular regulatory mechanisms of RIF and identifying key therapeutic targets hold significant clinical promise for overcoming the current impasse in preventing and treating chronic radiation‐induced intestinal damage.

Fibrosis is fundamentally characterized by the excessive deposition and aberrant cross‐linking of extracellular matrix (ECM) [8]. It is noteworthy that while ECM deposition is a necessary physiological process for tissue repair, its excessive accumulation leads to tissue stiffening and organ dysfunction [8]. Within this process, activated myofibroblasts serve as the principal effector cells responsible for ECM production. These cells predominantly originate from activated fibroblasts, and the degree and persistence of their activation directly dictate the severity of fibrosis [9]. Under the pathological conditions of radiation‐induced intestinal injury, luminal contents and diverse mediators create a complex microenvironment that crucially influences fibroblast activation and RIF progression [10, 11, 12]. These microenvironmental components play a decisive role in the activation of fibroblasts and the progression of RIF; for instance, genetic ablation of platelet‐derived growth factor C (PDGF‐C) has been shown to significantly suppress intestinal fibroblast activation [6]. Recently, metabolomics has offered a novel perspective for studying radiation injury. Ionizing radiation induces characteristic alterations in metabolic profiles [13, 14]. These differential metabolites not only hold potential as biomarkers but also actively participate in the pathological processes of radiation damage. For example, radiation exposure markedly reduces the level of indole‐3‐propionic acid in intestinal contents, and exogenous supplementation alleviates radiation damage [15]. Furthermore, radiation induces aberrant overexpression of very long‐chain acyl‐CoA dehydrogenase (VLCAD), which is closely associated with radiation‐induced cell death [16]. These findings strongly suggest that specific metabolites may play pivotal roles in RIF progression, potentially by modulating fibroblast activation status.

Intriguingly, tryptophan, an essential amino acid, serves not only as a fundamental building block for protein synthesis but also as the substrate for generating diverse bioactive molecules with critical functions [17, 18]. Free tryptophan is primarily metabolized through three pathways: approximately 5% via the serotonin pathway, less than 1% through the indole pathway, and over 95% via the kynurenine (Kyn) pathway [19]. Accumulating research highlights the central role of Kyn and its downstream metabolites (e.g., quinolinic acid) in immunometabolic regulation, with dysregulation of this pathway implicated in the pathogenesis of numerous diseases [20]. In cardiovascular contexts, Kyn has emerged as a novel biomarker of cardiovascular risk; its serum levels are significantly elevated in patients with hypertensive heart failure and promote ventricular remodeling [21]. Critically, within the gastrointestinal tract, this pathway is activated under inflammatory and injurious conditions. For instance, Kyn markedly accumulates in intestinal tissue following chemotherapy‐induced injury [22]. Moreover, elevated Kyn levels are well‐established features of inflammatory bowel disease (IBD), where they modulate immune responses and correlate with tissue damage [20, 23].

However, the role and regulation of the complete Kyn pathway in fibrotic intestinal disorders—particularly those driven by physical injury such as radiation—remain completely unknown. Building on evidence that Kyn accumulates in models of intestinal injury, we hypothesize that tryptophan metabolic reprogramming, specifically the aberrant activation of the Kyn pathway, is a key driver in the pathogenesis of RIF. We propose that within the fibrotic microenvironment, this pathway is fundamentally repurposed from its canonical immune‐modulatory role to directly activate stromal fibroblasts and drive pathological collagen deposition. This study aims to test this hypothesis and elucidate the underlying molecular mechanisms. Our findings will advance the understanding of RIF pathogenesis and provide a promising avenue for developing therapeutic strategies to mitigate RIF.

## 2 Results

### 2.1 Ionizing Radiation Induces Long‐Term Elevation of Kyn Levels in Intestinal Tissue and Its Association with Fibrosis

**FIGURE 1:**
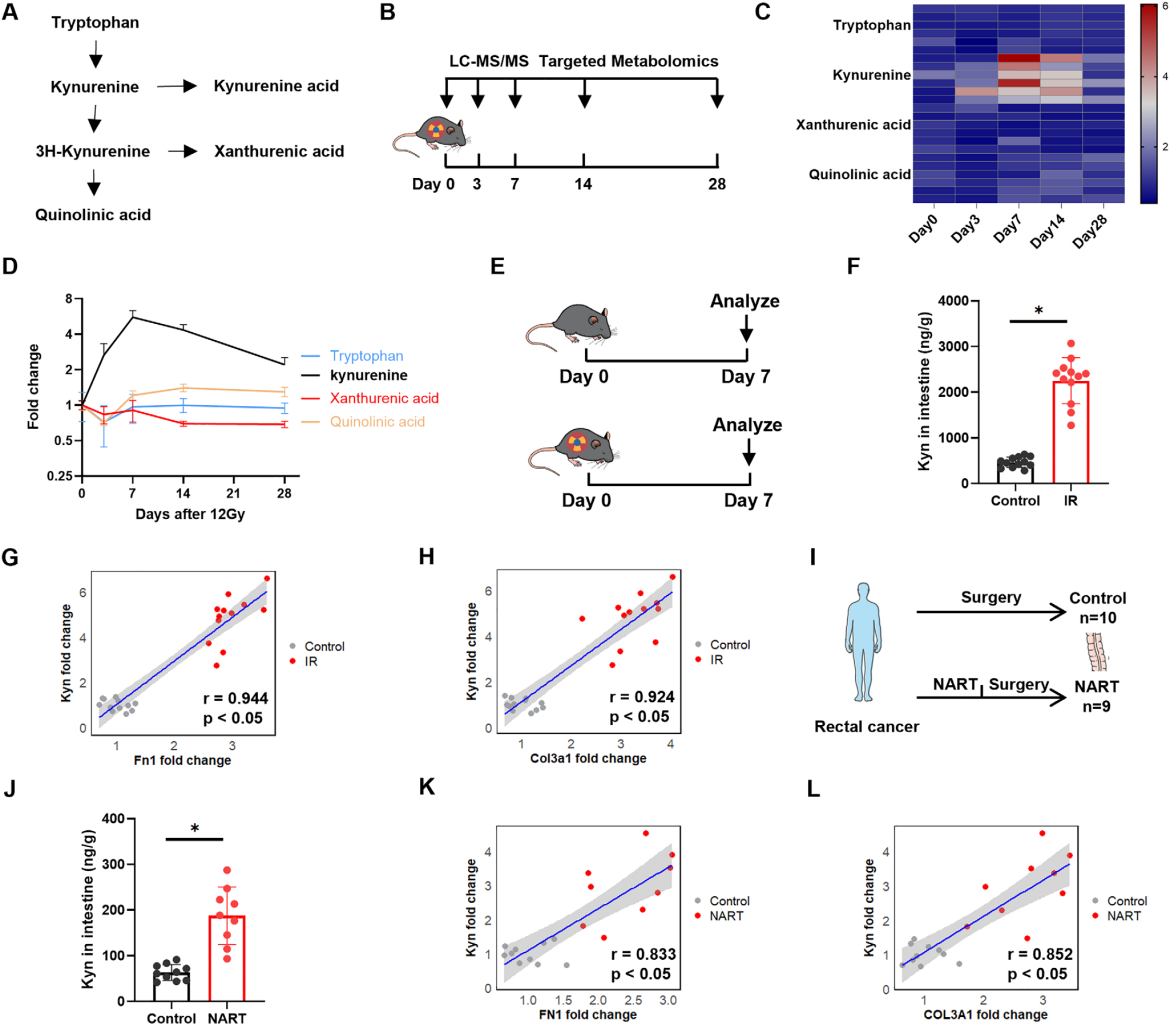
IR elevates Kyn levels in intestinal tissue. A) Kynurenine pathway. B) Mice received 12 Gy abdominal IR. C,D) Dynamic of Kyn pathway metabolites in intestinal tissue after IR (*n* = 6). E) Experimental scheme for (F,G). Mice received 12 Gy abdominal IR, and intestinal tissues are collected for analysis on day 7 post‐injection (*n* = 12). F) Kyn levels in intestinal tissue measured by ELISA at day 7 post‐IR (*n* = 12). G,H) Spearman correlation analysis for Kyn levels and the mRNA expression of Fn1 and Col3a1 in mouse intestinal tissue (*n* = 12). I) Clinical sample collection scheme. J) ELISA quantification of Kyn levels in intestinal tissue from clinical samples (*n* = 9–10). K,L) Spearman correlation analysis for Kyn levels and the mRNA expression of FN1 and COL3A1 in human intestinal tissue (*n* = 9–10). Data are presented as mean ± SD. *p < 0.05.

RIF exhibits progressive pathological characteristics. Consistent with previous studies [24], significant RIF pathological features, including submucosal thickening and increased collagen deposition, were evident in mouse intestines as early as 28 d following 12 Gy abdominal ionizing radiation (IR) (Figure S1A–C). The Kyn pathway (Figure 1A) of tryptophan is recognized as being involved in fibrotic diseases. To clarify the specific roles of Kyn pathway metabolites in RIF, we conducted a time‐course targeted metabolomic analysis in a murine model of abdominal IR (Figure 1B). We found that Kyn levels increased rapidly post‐IR, peaked at day 7, and remained elevated at approximately twice the baseline level at day 28 (Figure 1C,D; Table S1). This sustained elevation pattern closely mirrored the progressive nature of intestinal fibrosis. To validate these findings, we expanded the sample size (*n* = 12 per group) (Figure 1E) and quantified intestinal Kyn levels using ELISA, which confirmed a significant increase in intestinal Kyn levels at day 7 post‐IR (Figure 1F), highly consistent with the metabolomics data. Notably, Kyn levels showed significant positive correlations with the mRNA expression of fibrosis‐related genes Fn1 and Col3a1 in intestinal tissue (Figure 1G,H). These results suggest a potential causal role for Kyn in the initiation and progression of RIF.

To explore the potential clinical relevance of the animal findings, we further analyzed relevant clinical samples. We examined intestinal tissue samples from 10 rectal cancer patients without neoadjuvant therapy and 9 patients who received neoadjuvant radiotherapy (Figure 1I; Table S2). Kyn levels were significantly higher in the intestinal tissue of irradiated patients compared to the non‐irradiated group (Figure 1J). Furthermore, intestinal Kyn levels also demonstrated significant positive correlations with the mRNA expression of FN1 and COL3A1 (Figure 1K,L), supporting the pathway's clinical relevance. To directly link Kyn accumulation to histopathological fibrosis, we performed Masson's trichrome staining and COL3A1 immunohistochemistry on the same clinical sections. Tissues from radiotherapy patients showed marked submucosal thickening and collagen deposition (Figure S1D–F). Quantitatively, tissue Kyn concentration positively correlated with the severity of submucosal fibrosis, as measured by submucosal thickness (Figure S1G). These data provide direct histopathological evidence in human RIF that Kyn levels are associated with fibrotic tissue remodeling. In conclusion, data from both animal models and clinical samples indicate that Kyn is a key mediator likely involved in the pathogenesis and progression of RIF.

### 2.2 Kyn Exacerbates RIF

**FIGURE 2:**
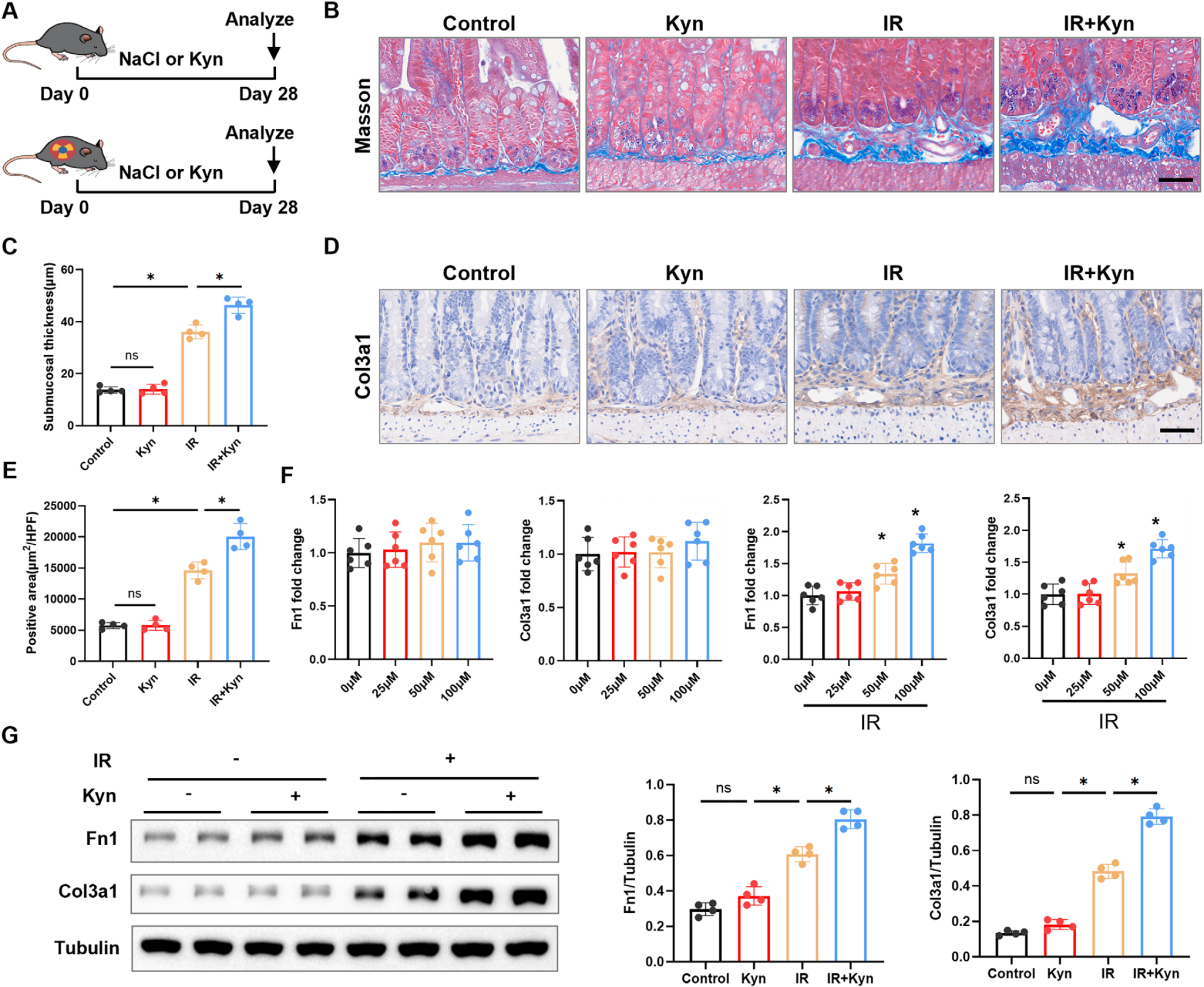
kyn exacerbates RIF. A) Experimental scheme for (B–E). Mice receive daily intraperitoneal injections of Kyn, and intestinal tissues are collected for analysis on day 28 post‐injection (*n* = 6). B) Representative Masson's trichrome staining of small intestinal tissue (scale bar = 50 µm). C) Quantitative analysis of intestinal submucosal thickness (*n* = 4). D) Representative COL3A1 staining of small intestinal tissue (scale bar = 50 µm). E) Quantitative analysis of COL3A1‐positive area in the submucosa (*n* = 4). F) Effect of Kyn treatment on mRNA expression of Fn1 and Col3a1 in primary intestinal fibroblasts (*n* = 6). G) Effect of Kyn treatment on protein expression of FN1 and COL3A1 in primary intestinal fibroblasts (*n* = 4). Data are presented as mean ± SD. *p < 0.05.

To evaluate the role of Kyn in RIF, we administered intraperitoneal injections of Kyn (50 mg kg^−1^) to mice (Figure 2A). This dosage was selected based on its established efficacy in promoting pathological remodeling in other disease models [21] and was validated in our system: a single injection of 50 mg kg^−1^ Kyn in naïve mice elevated intestinal Kyn concentration to approximately 1800 ng g^−1^ at 6 h postinjection, a level comparable to the endogenous concentration observed on day 14 post‐IR (Figure S2A and Table S1). Following 4 weeks of intervention, mice in the IR + Kyn group exhibited significantly aggravated intestinal damage compared to the IR alone group, characterized by more pronounced submucosal thickening (Figure 2B,C). Immunohistochemical staining further revealed significantly higher expression of the fibrosis marker protein COL3A1 in the small intestine of the IR + Kyn group compared to the IR group (Figure 2B,D). Furthermore, we isolated primary mouse intestinal fibroblasts. Under IR treatment, Kyn upregulated the mRNA expression of fibrosis‐related genes Fn1 and Col3a1 in these fibroblasts in a dose‐dependent manner. The maximum effect was observed at 100 µm (Figure 2E), which was subsequently selected for further cellular experiments. Western blot analysis confirmed that Kyn significantly upregulated FN1 and COL3A1 protein expression levels under IR conditions (Figure 2F). To investigate whether Kyn might exacerbate fibrosis indirectly by inducing epithelial‐mesenchymal transition (EMT), we next examined its effect on intestinal epithelial cells. we treated intestinal epithelial (IEC6) cells with Kyn and/or IR. IR alone induced EMT (reduced E‐cadherin, increased Vimentin), but the addition of Kyn did not further enhance these changes (Figure S2B,C). This suggests that Kyn does not exacerbate fibrosis by augmenting EMT in intestinal epithelial cells. Collectively, these results demonstrate that Kyn exacerbates intestinal fibrosis in the context of IR.

### 2.3 Kyn Promotes RIF by Activating the AHR

**FIGURE 3:**
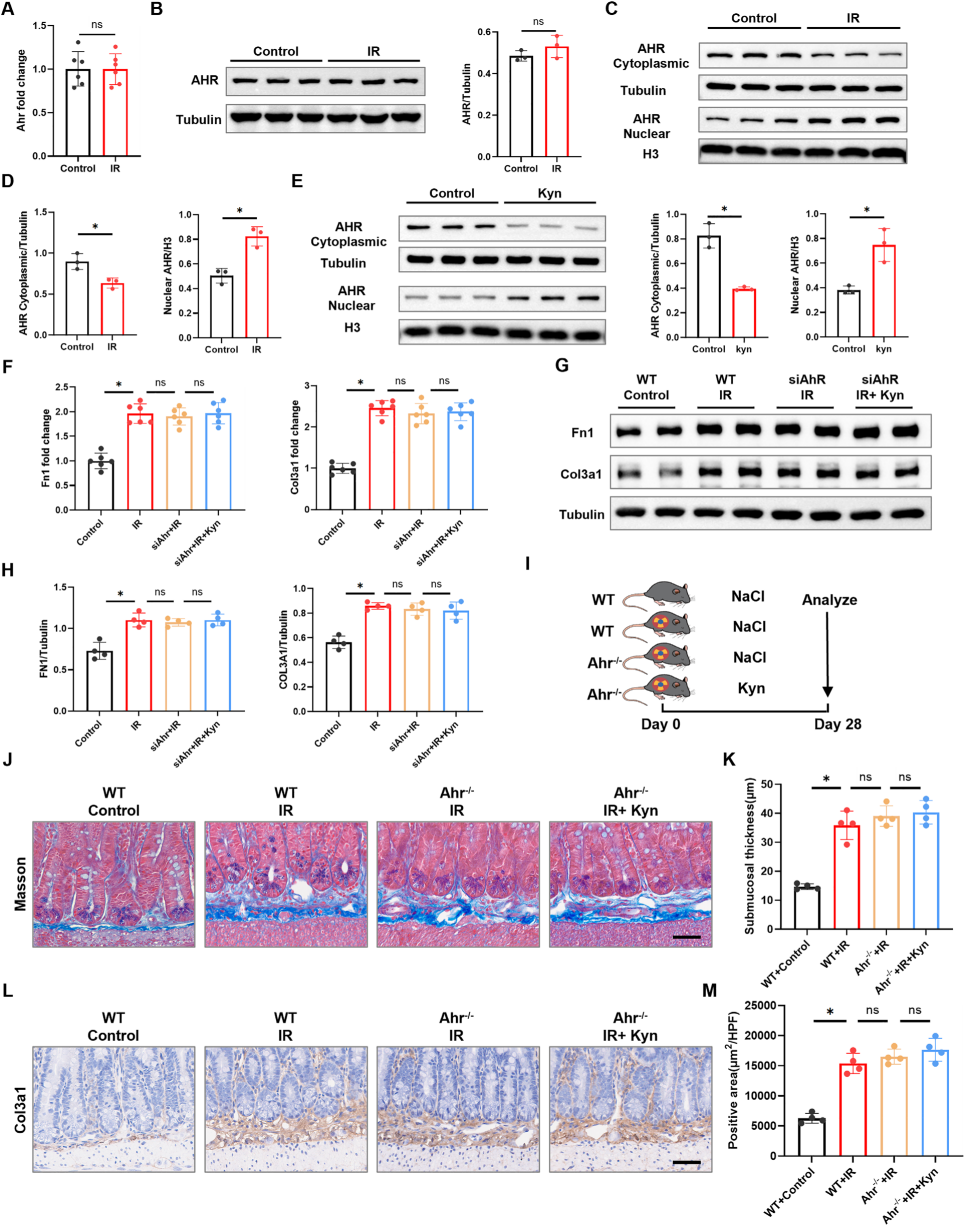
AHR mediates Kyn‐induced promotion of RIF. A) qRT‐PCR analysis of Ahr mRNA expression in control and IR‐treated intestinal fibroblasts (*n* = 6). B) Western blot analysis of total AHR protein expression in control and IR‐treated intestinal fibroblasts (*n* = 3). C,D) Western blot analysis of AHR protein distribution in cytoplasmic and nuclear fractions of intestinal fibroblasts from control and IR‐treated mice (*n* = 3). E) Western blot analysis of AHR protein distribution in cytoplasmic and nuclear fractions of intestinal fibroblasts treated with Kyn (*n* = 3). F) qRT‐PCR analysis of the effect of Kyn on mRNA expression of Fn1 and Col3a1 in intestinal fibroblasts following Ahr knockdown (*n* = 6). G,H) Western blot analysis of the effect of Kyn on protein expression of FN1 and COl3A1 in intestinal fibroblasts following Ahr knockdown (*n* = 3). I) Experimental scheme for (J–M) (*n* = 6). J) Representative Masson's trichrome staining of small intestinal tissue (scale bar = 50 µm). K) Quantitative analysis of intestinal submucosal thickness (*n* = 4). L) Representative COL3A1 staining of small intestinal tissue (scale bar = 50 µm). M) Quantitative analysis of COL3A1‐positive area in the intestinal submucosa (*n* = 4). Data are presented as mean ± SD. *p < 0.05.

Next, we investigated the mechanism by which Kyn promotes RIF. Given that Kyn is a potent endogenous ligand of the aryl hydrocarbon receptor (AHR) [25], we examined AHR activation in intestinal fibroblasts. Primary intestinal fibroblasts were isolated from control mice and IR‐treated mice. While no significant differences were observed in total Ahr mRNA (Figure 3A) or total AHR protein expression (Figure 3B) between control and IR‐treated groups, indicating that IR itself does not directly regulate Ahr expression. However, subcellular fractionation revealed a critical alteration: IR‐treated cells exhibited decreased cytoplasmic AHR distribution concomitant with increased nuclear accumulation (Figure 3C,D), demonstrating AHR activation via nuclear translocation. To determine whether radiation‐induced AHR translocation is mediated by Kyn, we exogenously treated primary intestinal fibroblasts with Kyn in vitro. This treatment recapitulated the IR‐induced phenotype, reducing cytoplasmic AHR while enhancing its nuclear localization (Figure 3E). To further validate Kyn‐induced AHR activation, we performed immunofluorescence staining, which confirmed the nuclear translocation of AHR upon Kyn treatment (Figure S3A). Furthermore, Kyn treatment significantly upregulated the mRNA expression of the AHR target gene Cyp1b1 (Figure S3B). Finally, co‐immunoprecipitation assays demonstrated that Kyn treatment increased the poly‐ubiquitination of AHR (Figure S3C), a hallmark of ligand‐induced AHR activation. Together, these data provide multifaceted evidence that Kyn acts as a functional ligand to activate the AHR signaling pathway.

To determine the role of AHR in mediating Kyn's pro‐fibrotic effects in vitro, we knocked down Ahr in mouse intestinal fibroblasts (Figure S3D). This knockdown significantly blocked the upregulation of mRNA and protein expression of the fibrosis‐related genes Fn1 and Col3a1 induced by Kyn supplementation in the context of IR, as assessed by comparing the siAhr+IR and siAhr+Kyn+IR groups (Figure 3F–H). Intriguingly, Ahr knockdown did not reverse the upregulation of these fibrotic markers induced by IR alone (Figure 3F–H, IR vs siAhr+IR). This suggested that the pro‐fibrotic effect of AHR activation was contingent upon the availability of its ligand, Kyn. To test this, we measured Kyn concentrations in the culture system. As shown in Figure S3G, endogenous Kyn levels were negligible in fibroblasts subjected to IR alone, whereas exogenous Kyn supplementation achieved a high micromolar concentration. Thus, IR alone can activate fibroblasts through AHR‐independent pathways, while the Kyn‐AHR axis becomes a major driver specifically under conditions of elevated Kyn availability.

To validate this finding in vivo, we employed an Ahr‐knockout mouse model (Figure 3I and Figure S3E,F). Results showed that in the absence of Ahr, IR + Kyn treatment did not significantly increase intestinal submucosal thickness (Figure 3J,K) or COL3A1 expression levels (Figure 3L,M) compared to IR treatment alone. Taken together, these in vitro and in vivo findings consistently demonstrate that AHR is the critical receptor molecule mediating Kyn's pro‐fibrotic effects in RIF.

### 2.4 Radiation Induces IDO1 Expression, Leading to Elevated Kyn

**FIGURE 4:**
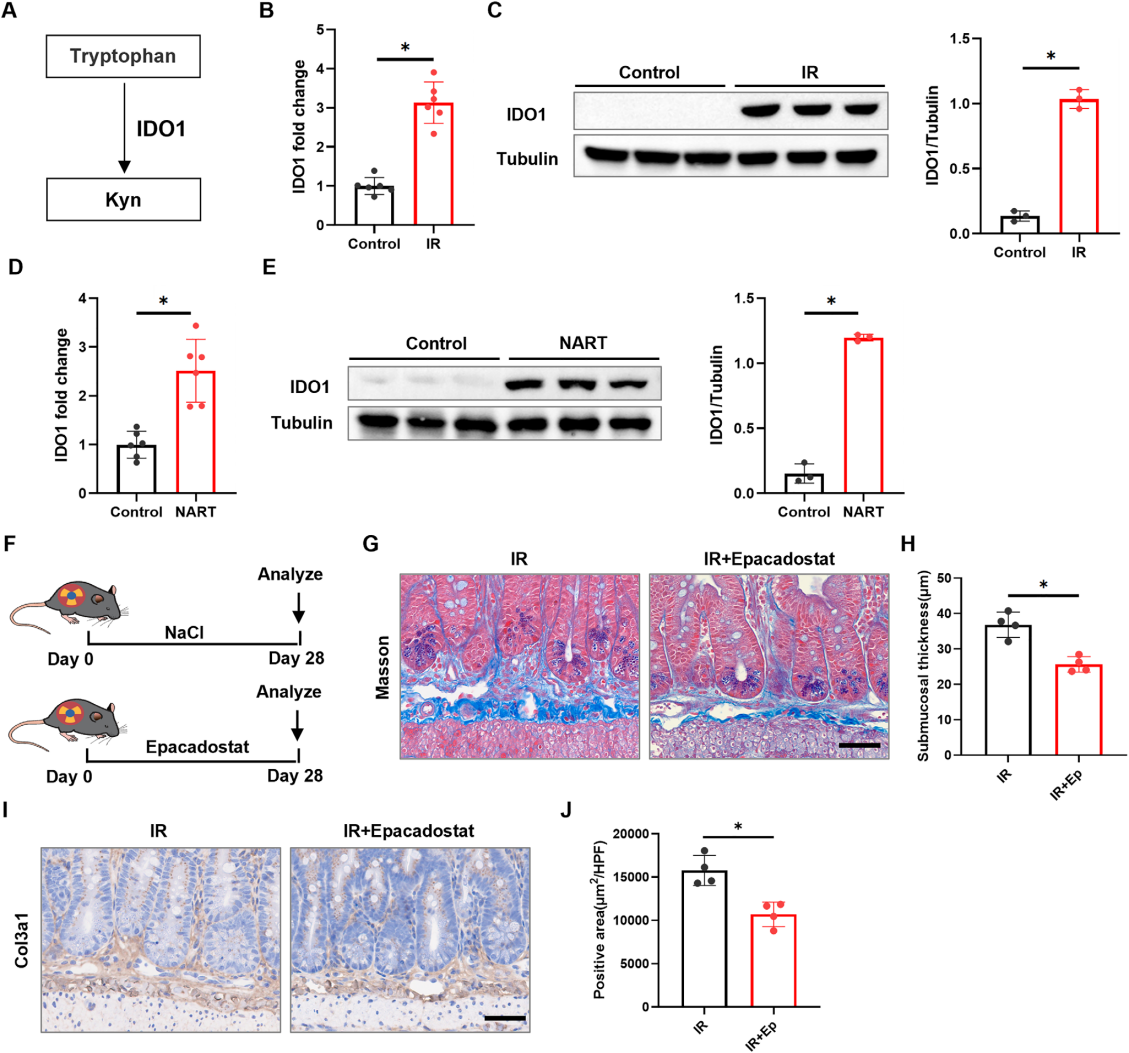
IR upregulates IDO1 to promote kyn production. A) Kyn pathway. B) Effect of IR on mRNA expression of IDO1 in mice intestinal tissue (*n* = 6). C) Effect of IR on mRNA and protein expression of IDO1 in mice intestinal tissue (*n* = 4). D) Effect of IR on mRNA expression of IDO1 in human intestinal tissue (*n* = 6). E) Effect of IR on protein expression of IDO1 in human intestinal tissue (*n* = 3). F) Experimental scheme for (G–J). Mice received Epacadostat to inhibit Kyn production (*n* = 6). G) Representative Masson's trichrome staining of small intestinal tissue (scale bar = 50 µm). H) Quantitative analysis of intestinal submucosal thickness (*n* = 4). I) Representative COL3A1 staining of small intestinal tissue (scale bar = 50 µm). J) Quantitative analysis of COL3A1‐positive area in the intestinal submucosa (*n* = 4). Data are presented as mean ± SD. *p < 0.05.

The key rate‐limiting enzyme responsible for catalyzing the conversion of tryptophan to Kyn is indoleamine 2,3‐dioxygenase 1 (IDO1), as illustrated in Figure 4A [17]. We first confirmed that IR significantly upregulated IDO1 at both mRNA and protein levels in murine and human intestinal tissue homogenates (Figure 4B–E). Immunofluorescence co‐staining on intestinal sections identified the intestinal epithelium (co‐localized with EPCAM) as a major site of IDO1 upregulation post‐IR, with minimal contribution from CD45^+^ leukocytes or CD11c^+^ dendritic cells (Figure S4A–C). Additionally, IDO1 was detected in non‐epithelial, non‐leukocyte stromal cells within the submucosa (Figure S4A–C). These results demonstrate that radiation induces IDO1 expression in multiple cellular compartments, with epithelial cells being a predominant source.

To further assess the functional role of IDO1 activation in RIF, we administered the selective IDO1 inhibitor Epacadostat [26] to a mouse model (Figure 4F). Subsequent histological evaluation using Masson's trichrome staining showed that Epacadostat treatment significantly attenuated IR‐induced submucosal thickening (Figure 4G,H), suggesting a reduction in collagen deposition and structural remodeling. Consistent with these findings, immunohistochemical analyses for COL3A1 demonstrated that Epacadostat effectively suppressed the increase in COL3A1 expression elicited by radiation (Figure 4I,J). Consistent with the histological findings, Western blot analysis confirmed that Epacadostat treatment significantly reduced the protein levels of FN1 and COL3A1 in intestinal tissues (Figure S4D,E).

To explore the tissue specificity of this pathway, we established a radiation‐induced pulmonary fibrosis model (12 Gy thoracic irradiation) and treated mice with Epacadostat (Figure S4F). IDO1 expression and Kyn levels were elevated in irradiated lungs at day 7 after IR, and Epacadostat treatment specifically suppressed the Kyn increase (Figure S4G–I). However, this inhibition did not significantly attenuate the late‐stage upregulation of fibrotic markers Col3a1 and Fn1 mRNA or reduce collagen deposition at day 28 post‐IR (Figure S4J–L). Taken together, these data support a critical contribution of the IDO1‐Kyn‐AHR axis to the pathogenesis of RIF. The lack of effect in the lung model suggests that the importance of this metabolic pathway may vary across different fibrotic tissues.

### 2.5 IR Regulates the IDO1‐kyn Metabolic Axis Partly via the Gut Microbiota

**FIGURE 5:**
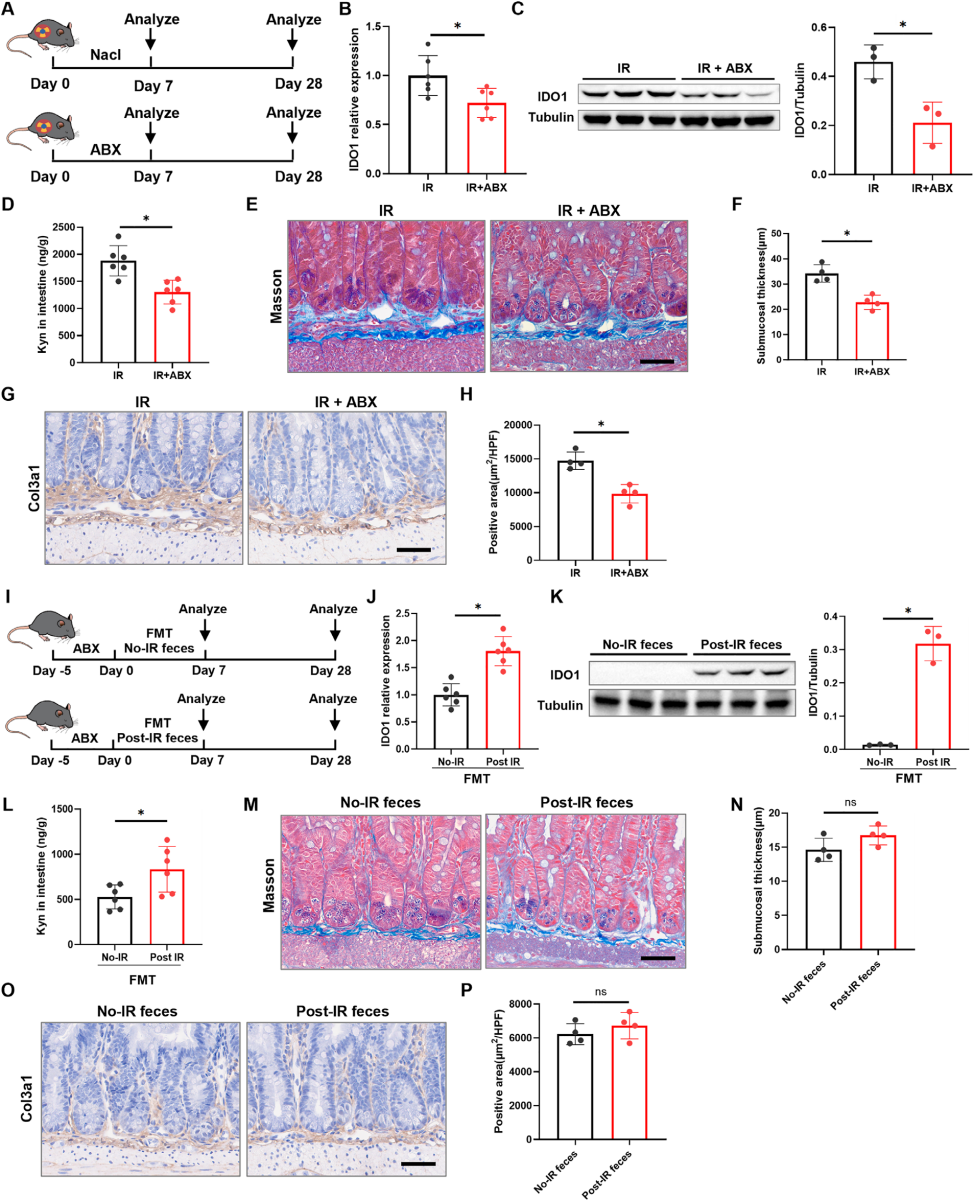
The intestinal IDO1‐Kyn metabolic axis is regulated by the gut microbiota. A) Experimental scheme for (B–G). Mice received broad‐spectrum antibiotic cocktail (ABX) for 7 d to deplete the gut microbiota (*n* = 6). B) qRT‐PCR analysis of IDO1 mRNA levels in intestinal tissues (*n* = 6). C) Western blot analysis of IDO1 protein levels in intestinal tissues (*n* = 3). D) ELISA detection of Kyn levels in intestinal tissues (*n* = 6). E) Masson staining of small intestinal tissue (scale bar = 50 µm). F) Quantitative analysis of intestinal submucosal thickness (*n* = 4). G) COL3A1 staining of small intestinal tissue (scale bar = 50 µm). H) Quantitative analysis of COL3A1‐positive area in the intestinal submucosa (*n* = 4). I) Experimental scheme for (I–N). Antibiotic‐treated mice underwent fecal microbiota transplantation (FMT) using post‐IR feces or feces from control mice (*n* = 6). J) qRT‐PCR analysis of IDO1 mRNA levels in intestinal tissues 7 d after FMT (*n* = 6). K) Western blot analysis of IDO1 protein levels in intestinal tissues 7 d after FMT (*n* = 3). L) ELISA detection of Kyn levels in intestinal tissues 7 d after FMT (*n* = 6). M) Masson staining of small intestinal tissue 28 d after FMT (scale bar = 50 µm). N) Quantitative analysis of intestinal submucosal thickness (*n* = 4). O) COL3A1 staining of small intestinal tissue 28 d after FMT (scale bar = 50 µm). P) Quantitative analysis of COL3A1‐positive area in the intestinal submucosa (*n* = 4). Data are presented as mean ± SD. *p < 0.05.

Building upon our findings that Kyn activates AHR signaling to promote RIF, we further investigated the mechanism by which IR regulates the IDO1‐Kyn metabolic axis. Given our previous discovery and literature reports that IR induces significant gut microbiota dysbiosis [27] and the established link between RIF and gut microbiota [28], we hypothesized that IR‐induced alterations in the gut microbial community serve as a key mediator driving the activation of the IDO1‐Kyn axis. To test this hypothesis, we first depleted the gut microbiota in mice using a broad‐spectrum antibiotic cocktail (ABX) (Figure 5A). Compared to the control group, ABX‐treated mice exhibited significantly downregulated IDO1 expression (Figure 5B,C) and a marked reduction in Kyn concentration (Figure 5D) in intestinal tissues. Critically, at 28 days post‐IR, ABX treatment significantly attenuated IR‐induced pathological changes, evidenced by reduced intestinal submucosal thickness (Figure 5E,F) and a decreased area of COL3A1‐positive staining (Figure 5G,H) compared to irradiated controls. This was further supported by a significant decrease in FN1 and COL3A1 protein expression as shown by Western blot (Figure S5A,B).

To directly assess the capacity of IR‐perturbed microbiota to regulate host metabolism, we performed fecal microbiota transplantation (FMT) from either IR‐treated mice or control mice into ABX‐pretreated recipient mice (Figure 5I). Seven days post‐FMT, recipients of microbiota from IR mice (Post‐IR) showed significantly upregulated IDO1 expression (Figure 5J,K) and elevated Kyn levels (Figure 5L) in intestinal tissues compared to recipients of control microbiota (No‐IR). However, at 28 days post‐FMT, no significant differences were observed between the Post‐IR and No‐IR groups in either intestinal submucosal thickness (Figure 5M,N) or the area of COL3A1 staining (Figure 5O,P). Western blot analysis further supported this observation by showing no change in FN1 and COL3A1 protein levels (Figure S5C,D). Collectively, these results demonstrate that IR‐induced gut microbiota dysbiosis is sufficient to activate host intestinal IDO1 expression and drive Kyn production, establishing the gut microbiota as a critical regulator of the IDO1‐Kyn metabolic axis in response to IR.

### 2.6 Reduced Abundance of *P. coprophilus* Partially Mediates Radiation‐Induced Activation of the IDO1‐Kyn Axis

**FIGURE 6:**
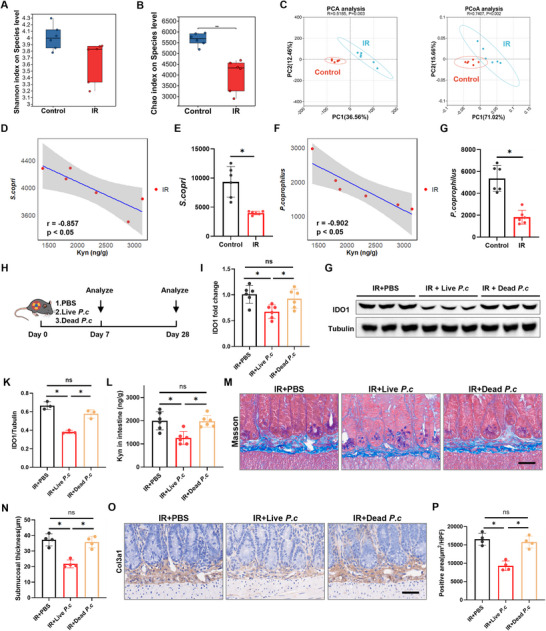
IR‐induced depletion of *P. coprophilus* partially mediates activation of the IDO1‐Kyn axis. A) Shannon analysis of alpha diversity (*n* = 6). B) Chao analysis of alpha diversity (*n* = 6). C) PCA and PCoA of fecal microbiota from control and IR mice (*n* = 6). D) Spearman correlation analysis between fecal *S. copri* abundance and intestinal tissue Kyn concentration in the IR group (*n* = 6). E) Relative abundance of *S. copri* in feces (*n* = 6). F) Spearman correlation analysis between fecal *P. coprophilus* abundance and intestinal tissue Kyn concentration in the IR group (*n* = 6). G) Relative abundance of *P. coprophilus* in feces (*n* = 6). H) Experimental scheme for (I–P). Intervention with live or inactivated *P. coprophilus* (*n* = 6). I) qRT‐PCR analysis of IDO1 mRNA levels in intestinal tissues (*n* = 6). G, K) Western blot analysis of IDO1 protein levels in intestinal tissues (*n* = 3). L) ELISA detection of Kyn levels in intestinal tissues (*n* = 6). M) Masson staining of small intestinal tissue (scale bar = 50 µm). N) Quantitative analysis of intestinal submucosal thickness (*n* = 4). O) COL3A1 staining of small intestinal tissue (scale bar = 50 µm). P) Quantitative analysis of COL3A1‐positive area in the intestinal submucosa (*n* = 4). Data are presented as mean ± SD. *p < 0.05.

To elucidate how IR regulates the IDO1‐Kyn axis through specific gut microbes, we performed metagenomic sequencing analysis on the fecal microbiota of mice post‐IR. The alpha and beta diversities analysis revealed significant separation between the IR‐treated and control groups (Figure 6A–C), confirming substantial IR‐induced alterations in microbial community structure. Given that supplementing specific microorganisms holds greater clinical significance [21], we focused on identifying core species that were significantly reduced after IR exposure. We employed a two‐step prioritization strategy: first, identifying species whose abundance showed a significant (inverse) correlation with intestinal Kyn levels in the IR group; second, from this correlated pool, selecting the most abundant species for in vivo functional testing, as high‐abundance members are more likely to exert substantial ecological and functional influence. Among these Kyn‐correlated species, *Segatella copri* (*S. copri*) and *P. coprophilus* were the two most abundant species in the control microbiota (Figure 6D–G and Figure S6A–C), positioning them as prime candidates for functional validation. Notably, among the species significantly depleted after IR (*p* < 0.05), *Bacteroides.sp* exhibited the highest relative abundance (Figure S6D). However, its abundance did not show a significant correlation with Kyn levels in the IR group (Figure S6E,F). Therefore, we focused on species that passed the correlation filter.

To directly assess the functional roles of candidate microbes in regulating IDO1‐Kyn metabolism, we first conducted intervention experiments with the most abundant strain, *S. copri* (Figure S6G). Oral administration of either live or dead *S. copri* to irradiated mice revealed that, compared with PBS‐gavaged controls, neither treatment suppressed radiation‐induced upregulation of IDO1 expression nor reduced intestinal Kyn levels (Figure S6H–K). These findings suggest that *S. copri* does not directly influence the IDO1‐Kyn axis.

We then extended our investigation to the second most abundant strain, *P. coprophilus*. qRT‐PCR analysis confirmed successful engraftment, with fecal *P. coprophilus* abundance approximately 3‐fold higher in the supplementation group compared to the PBS control (Figure S6L). Compared to the PBS‐gavaged control group, supplementation with live *P. coprophilus* significantly suppressed IR‐induced upregulation of IDO1 expression (Figure 6I–K) and concurrently reduced intestinal Kyn concentration (Figure 6L). Consistent with the reduction in Kyn, live *P. coprophilus* supplementation also attenuated the activation of the AHR pathway in vivo. Analysis of primary intestinal fibroblasts isolated from these mice showed downregulated expression of Cyp1b1 (Figure S6M). Pathological assessment at 28 days post‐IR further demonstrated that live *P. coprophilus* intervention attenuated RIF, as evidenced by reduced intestinal submucosal thickness (Figure 6M,N) and a decreased area of COL3A1‐positive staining (Figure 6O,P). Quantitative protein analysis corroborated these findings, showing that live *P. coprophilus* supplementation significantly suppressed the upregulation of FN1 and COL3A1 induced by IR (Figure S6N,O). Collectively, these findings indicate that IR‐induced depletion of *P. coprophilus* abundance partially mediates the activation of the IDO1‐Kyn axis and subsequent RIF development. Mechanistically, the reduction in *P. coprophilus* abundance alleviates its suppressive effect on IDO1 expression, thereby promoting aberrant Kyn accumulation in intestinal tissues and ultimately driving the progression of RIF.

### 2.7 6‐Methyluracil Mediates the Protective Effect of *P. coprophilus* against RIF

**FIGURE 7:**
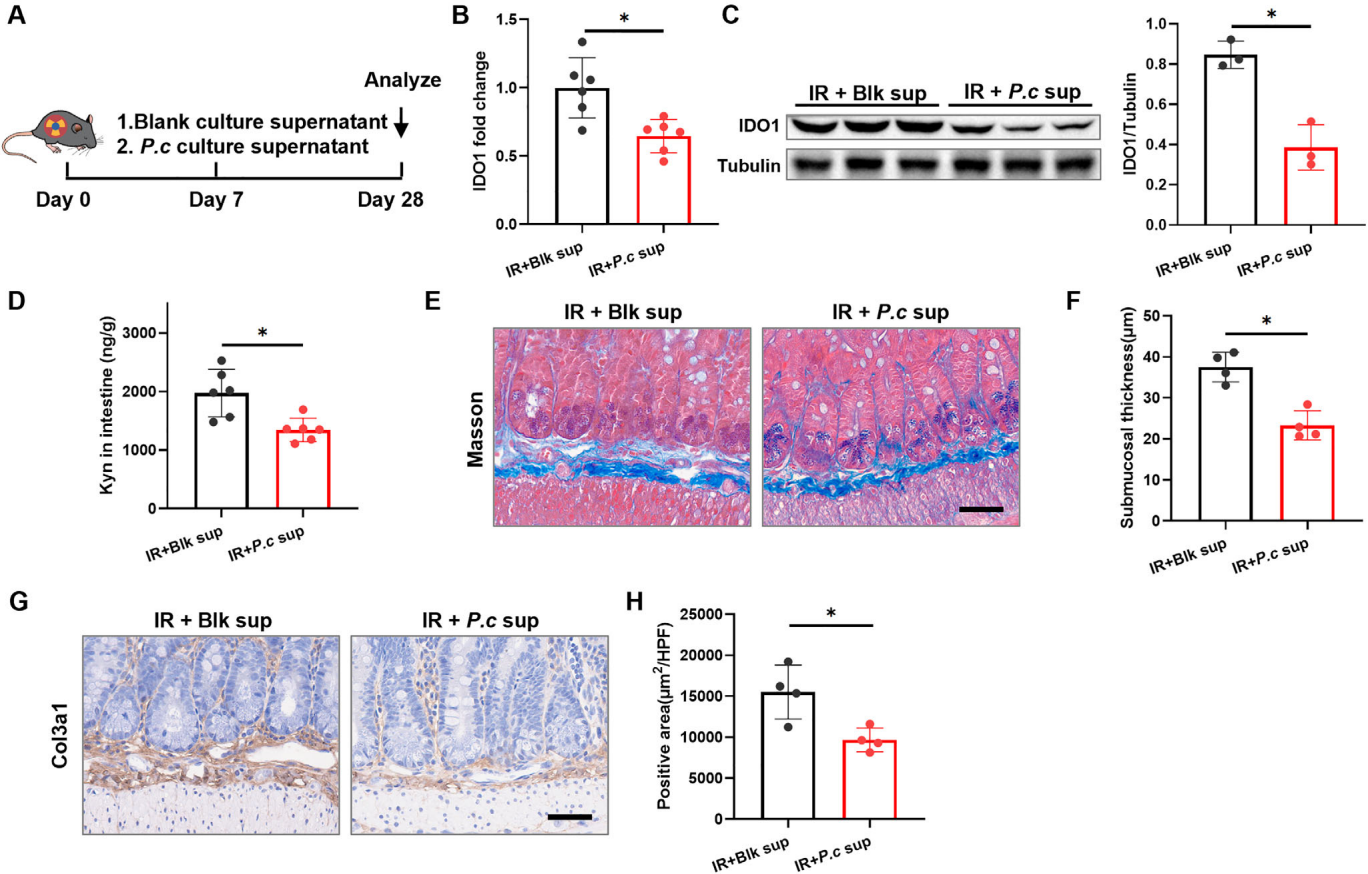
Metabolites of *P. coprophilus* alleviate RIF. A) Experimental scheme for (B–G). Mice were treated with *P. coprophilus* supernatant (*P. c* sup) or blank supernatant (Blk sup) (*n* = 6). B) qRT‐PCR analysis of IDO1 mRNA levels in intestinal tissues (*n* = 6). C) Western blot analysis of IDO1 mRNA and protein levels in intestinal tissues (*n* = 3). D) ELISA detection of Kyn levels in intestinal tissues (*n* = 6). E) Masson staining of small intestinal tissue (scale bar = 50 µm). F) Quantitative analysis of intestinal submucosal thickness (*n* = 4). G) COL3A1 staining of small intestinal tissue (scale bar = 50 µm). H) Quantitative analysis of COL3A1‐positive area in the intestinal submucosa (*n* = 4). Data are presented as mean ± SD. *p < 0.05.

Given that only live *P. coprophilus* effectively suppressed the IDO1‐Kyn axis and attenuated RIF, we investigated whether this protective effect is mediated by secreted bioactive compounds. To test this, irradiated mice were treated with the culture supernatant of *P. coprophilus* (*P. c* sup) (Figure 7A). The results showed that treatment with *P. c* sup significantly reduced intestinal IDO1 expression (Figure 7B,C), accompanied by a marked decrease in Kyn levels (Figure 7D) in irradiated mice, indicating potent suppression of the IDO1–Kyn pathway. Next, we performed detailed histopathological evaluations at day 28 post‐irradiation. Masson's trichrome staining revealed that *P. c* sup treatment substantially reduced submucosal thickening (Figure 7E,F). Consistent with this, immunohistochemical analysis for COL3A1 showed significantly lowered expression in the treatment group (Figure 7G,H). Western blot analysis confirmed the reduction in FN1 and COL3A1 protein levels in the *P. c* sup‐treated group (Figure S7A,B). Together, these results demonstrate that the antifibrotic benefits of *P. coprophilus* are mediated by secreted bioactive metabolite(s) present in the bacterial supernatant.

**FIGURE 8:**
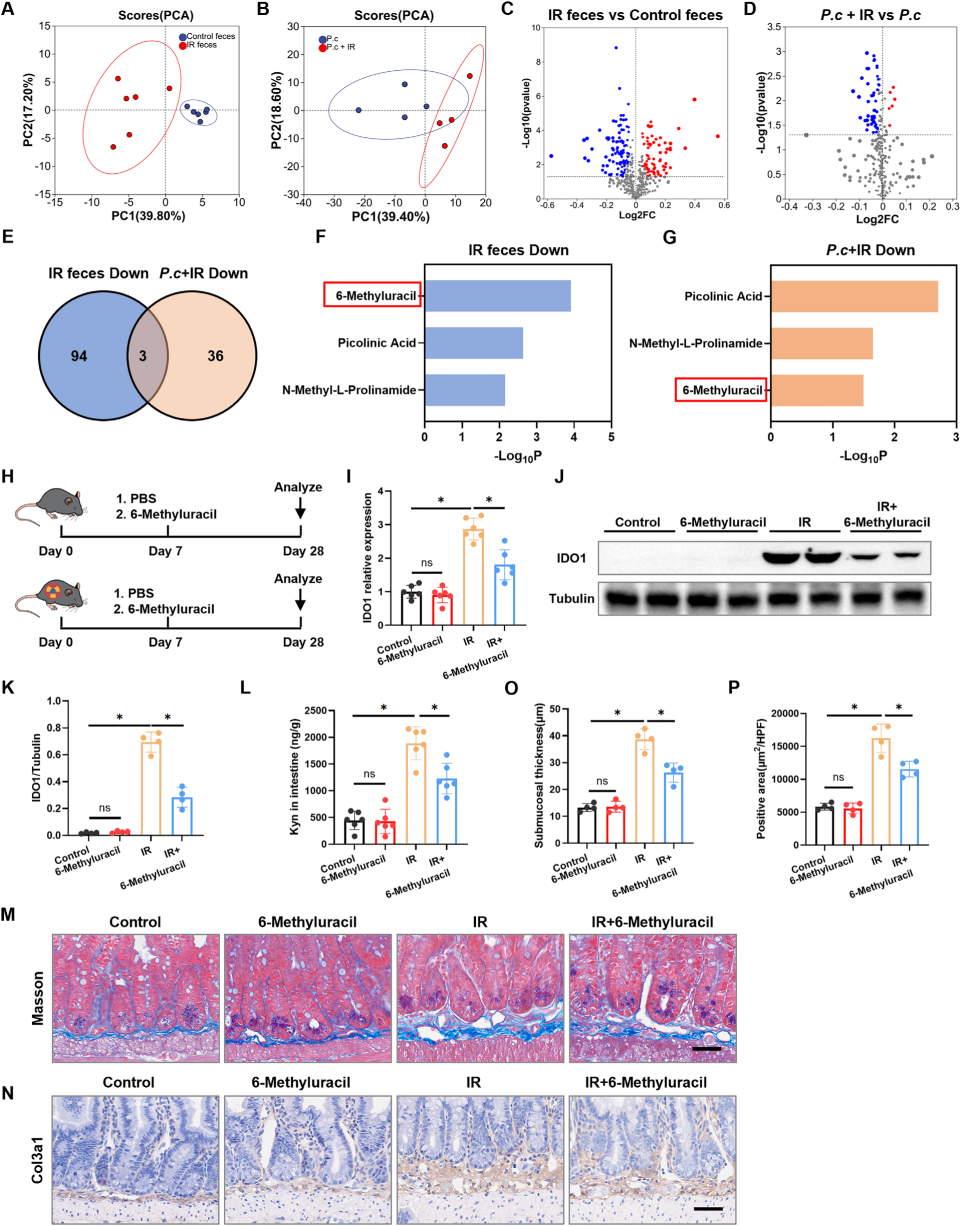
6‐Methyluracil alleviates RIF by inhibiting the IDO1‐Kyn axis. A) PCA of fecal metabolites from control and IR mice (*n* = 6). B) PCA of metabolites from *P. coprophilus* and *P. coprophilus*+IR (*n* = 6). C) Volcano plot showing differentially abundant fecal metabolites in IR‐treated mice (*n* = 6). D) Volcano plot showing differentially abundant metabolites in *P. coprophilus*+IR (*n* = 6). E) Venn diagram and bar chart showing commonly altered metabolites in feces from IR mice and *P. coprophilus* after IR. F,G) Three notable enriched metabolites in IR mice feces and *P. coprophilus*+IR were listed. H) Experimental scheme for (I–O). IR mice were treated with 6‐methyluracil (*n* = 6). I) qRT‐PCR analysis of IDO1 mRNA levels in intestinal tissues (*n* = 6). J,K) Western blot analysis of IDO1 protein levels in intestinal tissues (*n* = 4). L) ELISA detection of Kyn levels in intestinal tissues (*n* = 6). M) Masson staining of small intestinal tissue (scale bar = 50 µm). N) COL3A1 staining of small intestinal tissue (scale bar = 50 µm). O) Quantitative analysis of intestinal submucosal thickness (*n* = 4). P) Quantitative analysis of COL3A1‐positive area in the intestinal submucosa (*n* = 4). Data are presented as mean ± SD. *p < 0.05.

To identify the specific active metabolite responsible, we performed untargeted metabolomic profiling on fecal samples from post‐IR mice and on culture supernatants of *P. coprophilus* subjected to IR. Principal component analysis (PCA) and volcano analysis confirmed significant alterations in the metabolic profiles of both feces and *P. coprophilus* supernatants following IR exposure (Figure 8A–D). By cross‐comparing metabolites that were significantly reduced in both IR mouse feces versus control feces and in supernatants from IR‐treated *P. coprophilus* versus control *P. coprophilus*, we identified 6‐methyluracil as exhibiting the most pronounced decrease (Figure 8E–G), suggesting its potential role as a key regulator of the IDO1‐Kyn axis.

To functionally validate the role of 6‐methyluracil, we administered exogenous 6‐methyluracil to mice following IR exposure (Figure 8H). Compared to the IR‐only group, supplementation with exogenous 6‐methyluracil significantly suppressed intestinal IDO1 expression (Figure 8I–K) and reduced Kyn levels (Figure 8L). Consistent with this reduction in Kyn, 6‐methyluracil treatment also attenuated the activation of the AHR pathway in vivo. Primary intestinal fibroblasts isolated from 6‐methyluracil‐treated mice exhibited downregulated mRNA expression of Cyp1b1 (Figure S8A). Further pathological assessment at 28 d post‐IR, including Masson's trichrome and COL3A1 staining, revealed that 6‐methyluracil treatment attenuated submucosal thickening (Figure 8M,O) and suppressed COL3A1 expression (Figure 8N,P). The amelioration of fibrosis was further validated at the protein level, with 6‐methyluracil treatment significantly reducing the expression of FN1 and COL3A1 (Figure S8B,C).

To determine whether 6‐methyluracil directly acts on fibroblasts or antagonizes AHR signaling, we performed in vitro experiments. Treatment of primary intestinal fibroblasts with 6‐methyluracil under IR conditions did not directly suppress the upregulation of FN1 and COL3A1 protein levels (Figure S8D,E). Furthermore, 6‐methyluracil did not prevent Kyn‐induced AHR nuclear translocation or the upregulation of Cyp1b1 in cultured fibroblasts (Figure S8F–I). These in vitro data indicate that 6‐methyluracil does not function as a direct antagonist of fibroblast activation or the Kyn‐AHR axis within stromal cells. Instead, they support the model that its anti‐fibrotic effect in vivo is mediated primarily through its upstream action in suppressing the IDO1‐Kyn pathway.

To evaluate the translational potential of 6‐methyluracil in a more clinically relevant context, we tested its efficacy in MC38 colon cancer‐bearing mice subjected to abdominal irradiation. Due to the rapid progression of MC38 tumors, the observation window was limited to 7 d post‐irradiation to maintain animal welfare (Figure S9A); therefore, this experiment assessed early pathway activation rather than chronic fibrosis. In this model, radiation (but not the tumor itself) significantly activated the intestinal IDO1‐Kyn axis and upregulated Col3a1 and Fn1. Crucially, administration of 6‐methyluracil effectively suppressed this radiation‐specific activation (Figure S9B–F). These results demonstrate that the protective effect of 6‐methyluracil against the IDO1‐Kyn pathway is maintained in a tumor‐bearing host, underscoring its potential applicability for preventing RIF in cancer patients undergoing radiotherapy.

Taken together, these findings confirm that 6‐methyluracil, a metabolite derived from *P. coprophilus*, functions as a protective agent by inhibiting the IDO1–Kyn signaling pathway and ultimately attenuating RIF.

## 3 Discussion

**FIGURE 9:**
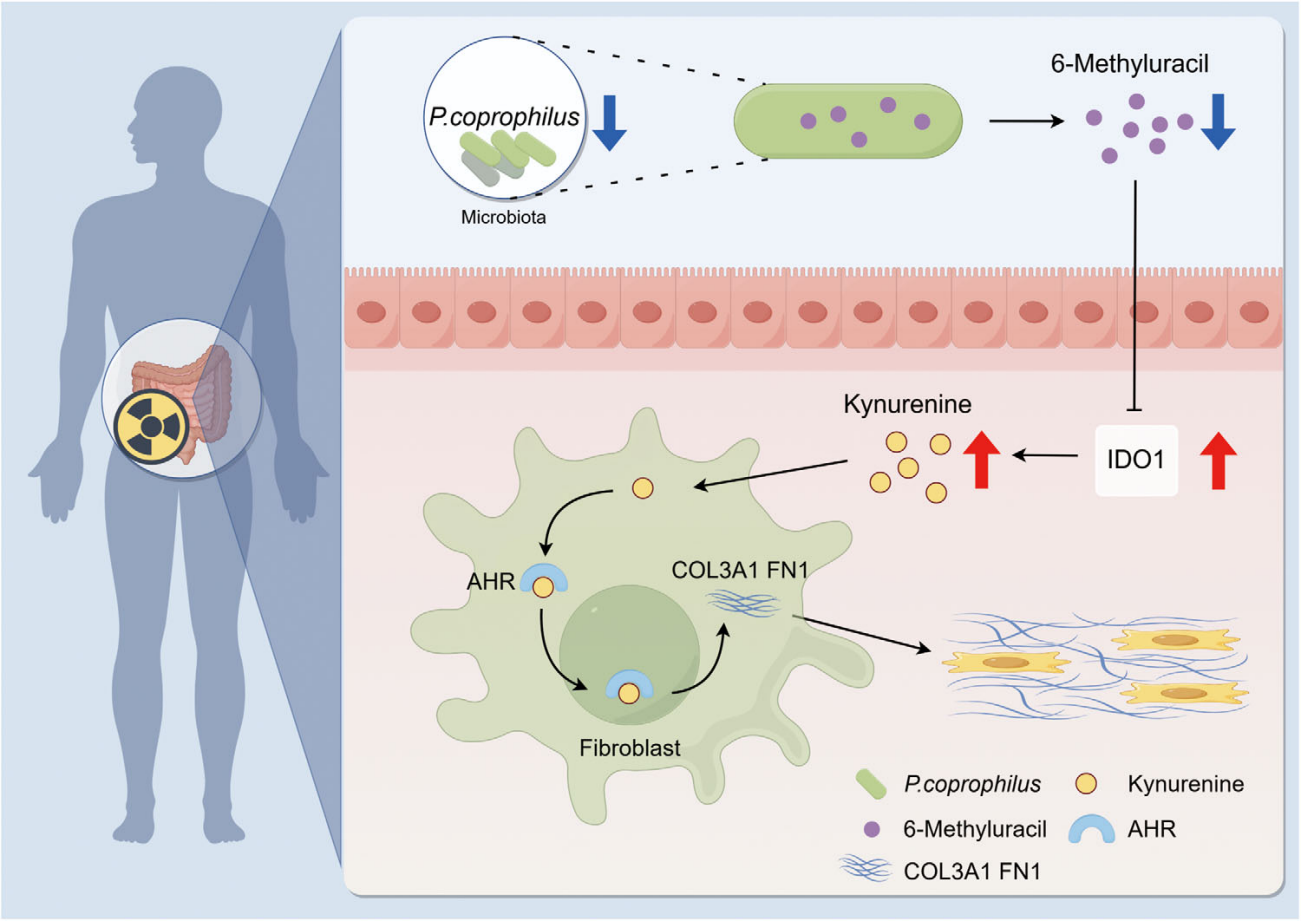
IR‐induced dysbiosis depletes *P. coprophilus* and its metabolite 6‐Methyluracil, leading to disinhibition of the IDO1‐Kyn‐AHR axis. This results in sustained fibroblast activation and collagen deposition, driving RIF.

RIF is a severe and progressive complication of abdominal radiotherapy, for which effective treatments remain limited. This study unveils a novel pathogenic axis driving RIF, centered on the tryptophan metabolite kyn. We demonstrate that IR triggers gut microbiota dysbiosis, leading to a depletion of *P. coprophilus* and its protective metabolite, 6‐methyluracil. This loss results in the derepression of the IDO1‐Kyn‐AHR signaling pathway, which we identify as a central driver of fibroblast activation and collagen deposition in the irradiated intestine (Figure 9).

Our findings are consistent with the growing recognition of metabolomics in identifying functional metabolites critical to disease pathogenesis [29, 30, 31, 32]. Kyn, a key intermediate in tryptophan metabolism, has been shown to be significantly upregulated in inflammatory models such as chemotherapy‐induced enteritis and DSS‐induced colitis [20, 22], with subsequent studies revealing its direct role in promoting pathological cardiac remodeling. Given the potential link of Kyn to inflammatory and fibrotic processes, this study focused on RIF. Integrating analyses from RIF animal models and patient samples, we confirmed for the first time that Kyn is significantly and persistently elevated in RIF. Critically, Kyn levels showed a significant positive correlation with the expression of key fibrosis markers in intestinal tissue, suggesting Kyn is not merely a bystander but an active contributor to the fibrotic cascade. Functional experiments unequivocally demonstrated that exogenous Kyn administration exacerbates RIF. Although other metabolites in the tryptophan pathway may play roles in intestinal inflammation [20, 23, 33, 34], our metabolomic analysis revealed that only Kyn exhibited a pattern of change highly consistent with fibrosis progression.

We further delineated the mechanistic pathway through which Kyn exerts its effects. Kyn is a well‐established endogenous ligand of the AHR [35]. Our data demonstrate that radiation‐ induced Kyn promotes AHR activation and nuclear translocation in intestinal fibroblasts, directly upregulating the transcription of pro‐fibrotic genes. Both in vitro knockdown and in vivo knockout studies confirmed that AHR is indispensable for mediating Kyn's pro‐fibrotic effects specifically under conditions of elevated Kyn availability. Importantly, our in vitro data revealed that the pro‐fibrotic role of AHR is ligand‐dependent: in the context of IR, exogenous Kyn robustly activated the AHR‐fibrosis axis, exogenous Kyn robustly activated the AHR‐fibrosis axis, whereas radiation alone induced fibroblast activation through AHR‐independent pathways when Kyn levels were low. This ligand‐dependent dichotomy refines the model of AHR involvement in RIF, positioning the Kyn‐AHR axis as a critical, context‐dependent signaling pathway that becomes a major driver specifically when tryptophan metabolism is skewed towards Kyn overproduction.

Notably, the role of Kyn‐AHR axis in fibrosis is highly context‐dependent, influenced by the specific ligand, cell type, tissue microenvironment, and disease etiology [36, 37, 38, 39, 40, 41]. This is exemplified by Kyn itself, which exerts anti‐fibrotic effects in dermal fibroblasts [42], contrasting with its pro‐fibrotic role we identified in intestinal fibroblasts. Our study further reveals that even within radiation‐induced injury, this axis is not universally pathogenic. It is a central driver in the intestine but non‐essential in the irradiated lung, where its pharmacological inhibition failed to attenuate fibrosis.

This profound tissue and context specificity likely stems from several factors. Firstly, tissue‐specific differences in AHR expression, co‐regulators, and downstream target genes can fundamentally alter the transcriptional response to AHR activation [43]. Secondly, the unique microenvironment of each organ dictates the dominant signaling networks. In dermal wound healing or the irradiated lung, other potent pathways (e.g., TGF‐β/Smad) may be the primary fibrotic drivers, either overriding the contribution of the Kyn‐AHR axis or even engaging AHR in a compensatory, anti‐fibrotic program [38]. In contrast, the irradiated intestine, with its dense microbiota and intense mucosal immune activity, creates a unique permissive niche where microbiota‐dependent dysregulation of the IDO1‐Kyn axis becomes a pivotal and dominant pro‐fibrotic signal. Finally, the net effect of AHR activation likely represents a balance between pro‐fibrotic and anti‐fibrotic signals [44]. In RIF, the radiation‐disrupted microenvironment may tip this balance overwhelmingly towards fibrosis promotion via the Kyn‐AHR axis in stromal fibroblasts. Our findings therefore position the IDO1‐Kyn‐AHR axis not as a universal mediator with a fixed role, but as a highly plastic signaling module whose functional output is decisively shaped by the tissue and disease context.

A key feature of this study is the IDO1 as a central regulator of Kyn production in response to radiation. Our results show that IR exposure significantly upregulates IDO1 expression in both mouse and human intestinal tissues, leading to increased Kyn production. Importantly, pharmacological inhibition of IDO1 with Epacadostat reduced radiation‐induced fibrosis, indicating that IDO1 activation is a critical step in the progression of RIF.

The identification of epithelial and stromal cells as primary sources of IDO1 upregulation is particularly significant, as these cells reside in the intestinal mucosal layer—the critical interface for host‐microbiota crosstalk [18]. This spatial localization prompted us to investigate whether the gut microbiota, a key regulator of the mucosal microenvironment, influences the IDO1‐Kyn axis. Our previous research and literature reports suggest that IR disrupts the gut microbiota [45], and tryptophan metabolism is closely linked to microbiota status [46]. Therefore, we hypothesized that IR‐induced gut dysbiosis plays a pivotal role in the activation of the IDO1‐Kyn axis. Experiments involving antibiotic‐mediated microbiota depletion confirmed a significant association between microbiota presence and IDO1 expression/Kyn levels. More importantly, FMT experiments directly demonstrated that recipient mice receiving microbiota from irradiated mice exhibited upregulated IDO1 expression and elevated Kyn levels. These findings underscore the importance of the gut microbiota in modulating host metabolism and suggest that microbiota‐derived signals are key mediators of the IDO1‐Kyn pathway in response to radiation [21, 47].

Our FMT experiments revealed a critical nuance: while the irradiated microbiota was sufficient to dysregulate the IDO1‐Kyn axis, it failed to induce fibrosis. This paradox, consistent with the inability of exogenous Kyn alone to drive fibrosis (Result 2.2), clarifies that activation of this metabolic axis is necessary but not sufficient for RIF. We therefore propose a “two‐hit” pathogenesis model [7]: the first hit is persistent radiation damage to the intestinal tissue (e.g., DNA damage, barrier disruption, ischemia), creating a pro‐fibrotic microenvironment; the second hit is microbiota‐dependent derepression of the IDO1‐Kyn‐AHR pathway, which provides the key metabolic signal for fibroblast activation. The dysbiotic microbiota thus primes the stroma, but fibrosis execution requires synergy with ongoing host injury. This model explains the therapeutic efficacy of targeting the microbial component to interrupt the pro‐fibrotic cascade.

Notably, we observed that exposure to IR caused a significant reduction in the abundance of *P. coprophilus*, a gut bacterium that is inversely correlated with Kyn levels. Supplementation with live *P. coprophilus* significantly downregulation of intestinal IDO1 expression, which led to a reduction in Kyn levels, ultimately resulting in the attenuation of RIF. These findings suggest that *P. coprophilus* plays a protective role against RIF, possibly through the modulation of the IDO1‐Kyn axis. This is the first study to link *P. coprophilus* to RIF, highlighting the potential of gut microbiota‐based therapies for managing fibrotic diseases.

A growing body of evidence highlights that gut microbial metabolites are key signaling molecules mediating interactions between microbial communities and the host [48, 49, 50, 51]. For example, p‐coumaric acid from *Blautia producta* alleviates hepatocyte death and liver injury by inhibiting ROS generation in hepatocytes [52]. This “microbe‐metabolite‐host protection” paradigm prompted us to explore whether *P. coprophilus* exerts its RIF‐mitigating effects via specific metabolites. Based on metabolomic analysis of fecal samples and *P. coprophilus* culture supernatants, we inferred that 6‐methyluracil—a key metabolite of *P. coprophilus*—might be the key mediator underlying this protective effect. Subsequent functional validation confirmed that exogenous supplementation of 6‐methyluracil effectively inhibited IDO1 expression and Kyn production, leading to improved RIF outcomes. These results suggest that 6‐methyluracil may be a novel therapeutic candidate for RIF, capable of suppressing the IDO1‐Kyn axis. Notably, we confirmed that its efficacy in suppressing this axis is maintained in a tumor‐bearing mouse model, underscoring its potential applicability within the complex microenvironment of cancer patients undergoing radiotherapy.

The efficacy of 6‐methyluracil against RIF is consistent with its documented radioprotective properties in other tissues, which have been attributed to broad antioxidant effects such as inhibiting lipid peroxidation [53]. However, our study reveals a distinct and more specific mechanism in the context of intestinal fibrosis. Beyond general antioxidant activity, we demonstrate that 6‐methyluracil functions as a signaling metabolite that directly targets the IDO1‐Kyn‐AHR pathway. Our in vitro data show that 6‐methyluracil does not directly inhibit pro‐fibrotic gene expression in intestinal fibroblasts nor does it act as a direct AHR antagonist. Instead, it acts upstream to suppress radiation‐induced IDO1 expression, thereby preventing the activation of this pro‐fibrotic cascade. This pathway‐specific inhibition distinguishes our findings from prior work on 6‐methyluracil's role in mitigating acute radiation damage and repositions it as a precise modulator of chronic fibrotic progression. While antioxidant effects may contribute to a protective milieu, our data—where 6‐methyluracil recapitulated the effect of live bacteria by downregulating the IDO1‐Kyn axis—strongly argue that this signaling intervention is the dominant anti‐fibrotic mechanism in our model. Thus, our work redefines 6‐methyluracil's action from a general radioprotectant to a specific regulator of a microbiota‐dependent fibrogenic pathway.

This study has limitations. First, the clinical validation cohort was relatively small. These human data should be considered preliminary and hypothesis‐generating. Larger, prospective studies specifically designed to correlate Kyn/IDO1 levels with directly assessed RIF severity in humans are necessary to confirm the clinical translational potential of this pathway. Second, the use of a global Ahr knockout model limits cell‐specific insights; future studies employing fibroblast‐specific conditional knockouts would help precisely elucidate the mechanism of the Kyn‐AHR axis in RIF. Third, while we identified intestinal epithelial cells as the predominant source of IDO1 upregulation, the relative contribution of other cellular compartments (such as stromal cells within the submucosa) to the overall Kyn pool and fibrotic progression remains to be determined. Cell‐type‐specific knockout models of IDO1 would be valuable in dissecting these contributions. Additionally, the rapid progression of tumors in our MC38‐bearing mouse model limited the observation window to 7 d post‐irradiation, preventing assessment of chronic fibrosis development in this setting.

Another important consideration is the irradiation regimen used in our study. To establish a robust RIF phenotype, we employed a classical mouse model with a single high‐dose (12 Gy) abdominal irradiation [24, 54, 55]. This regimen effectively induces fibrotic pathological changes that closely mimic human RIF and is widely utilized in preclinical studies [24, 54, 55]. However, we acknowledge that this single high‐dose regimen differs from fractionated, low‐dose radiotherapy protocols used in clinical practice (e.g., 45–50.4 Gy delivered in 25–28 fractions over 5–5.5 weeks for rectal cancer) [56]. To bridge this translational gap and enhance the clinical relevance of our findings, future studies will implement fractionated irradiation models (e.g., 2 Gy per fraction, delivered 5 times per week over several weeks). In these models, we will systematically verify whether the activation of the IDO1‐Kyn‐AHR axis, depletion of *P. coprophilus*, and reduction of 6‐methyluracil persist, and will evaluate the prophylactic or therapeutic potential of *P. coprophilus* and 6‐methyluracil in this context.

In conclusion, this study elucidates a comprehensive mechanistic pathway linking radiation injury to fibrosis: IR induces dysbiosis, resulting in the depletion of *P. coprophilus* and its bioactive metabolite 6‐methyluracil. This loss disinhibits the IDO1‐Kyn‐AHR axis, triggering sustained fibroblast activation and collagen deposition, key features of RIF. These findings not only deepen our understanding of the pathological mechanisms underlying RIF but also propose two promising therapeutic strategies: probiotic supplementation with *P. coprophilus* and direct intervention with 6‐methyluracil. These strategies offer novel, translatable approaches for mitigating RIF, with potential for clinical application in reducing fibrosis and improving patient outcomes following radiation therapy.

## 4 Experimental Section

### 4.1 Patients

This study enrolled rectal cancer patients aged 18 to 75 years, including two groups: those who had received neoadjuvant radiotherapy and those who had not. Patients with inflammatory bowel diseases (such as ulcerative colitis, Crohn's disease, etc.) and immune system‐related diseases (such as systemic lupus erythematosus, rheumatoid arthritis, etc.) were excluded. Following surgery, non‐tumorous rectal tissue samples were collected for analysis. All procedures were performed in compliance with relevant laws and institutional guidelines. Informed consent was obtained from all participants. The study has been approved by the Ethics Committee of the First Hospital of Jilin University (No, 2025‐MS‐154).

### 4.2 Animals

This study used 6‐ to 8‐week‐old male C57BL/6J wild‐type mice and Ahr knockout mice, all provided by Gempharmatech Co., Ltd. All mice were housed in SPF‐grade animal facilities, with environmental temperature, humidity, and light cycles complying with experimental animal management standards. Mice had free access to food and water during the feeding period. This experiment has been approved by the Animal Ethics Committee of the First Hospital of Jilin University (No, 2023‐0721) and was implemented in strict accordance with the Regulations for the Administration of Experimental Animals and relevant ethical guidelines.

### 4.3 Mouse Radiation

After intraperitoneal injection of tribromoethanol (Sigma) for anesthesia, the mice were fixed in a specialized mouse radiation cage once they entered a state of deep anesthesia. The head, neck, and thoracic regions were strictly shielded with lead plates, exposing only the abdominal area to be irradiated. A single local abdominal irradiation was performed using an X‐ray radiation system, with specific parameters as follows: radiation dose of 12 Gy, voltage of 160 kV, and current of 25 mA. For the thoracic irradiation model to induce pulmonary fibrosis, mice were anesthetized and their thoracic regions were exposed to a single dose of 12 Gy X‐ray irradiation using similar parameters, with the rest of the body shielded.

### 4.4 Mouse Kyn Intervention

This experiment consists of two groups: the positive intervention group and the negative intervention group. Mice in the forward intervention group received daily intraperitoneal injection of 50 mg kg^−1^ Kyn (Shanghai Macklin) immediately after irradiation for 4 weeks, and tissue samples were collected after the intervention. The negative intervention group was given 100 mg kg^−1^ Epacadostat (Shanghai Macklin) by daily gavage for 4 weeks, with tissue samples collected after the intervention.

### 4.5 Subcutaneous Tumor Model and Abdominal Irradiation

MC38 cells were subcutaneously injected into the right flank of C57BL/6J mice (1×106 cells per mouse). When tumor volumes reached approximately 150 mm^3^, mice were randomly divided into groups: control, tumor, tumor + IR, and tumor + IR + 6‐methyluracil (50 mg kg^−1^). For irradiation, tumor‐bearing mice were anesthetized and received a single dose of 12 Gy abdominal irradiation. Mice were sacrificed on day 7 after the initiation of irradiation, and the small intestine was collected for analysis.

### 4.6 Intestinal Microbiota Depletion

Intestinal microbiota depletion was performed using a mixed antibiotic solution: Mice were gavaged daily with a combination of antibiotics [vancomycin (100 mg kg^−1^), neomycin sulfate (200 mg kg^−1^), ampicillin (200 mg kg^−1^), metronidazole (200 mg kg^−1^)] once a day for 7 consecutive days.

### 4.7 The Fecal Microbiota Transplantation (FMT)

Fecal samples were collected from mice before irradiation and on day 7 post‐irradiation. The feces were mixed with PBS at a ratio of 125 mg feces / 1 mL PBS and vortex‐mixed thoroughly to prepare a fecal suspension. The suspension was then centrifuged, and the supernatant was collected as the bacterial suspension. Prior to FMT, all mice received antibiotic pretreatment to deplete their endogenous gut microbiota. Via gastric gavage, mice were administered 150 µL of the fecal supernatant daily for 7 consecutive days. Intestinal tissue samples were harvested from the mice on days 7 and 28 after the first gavage.

### 4.8 Culture of *Phocaeicola coprophilus* and *Segatella copri*

*Phocaeicola coprophilus* (*P. coprophilus*, BNCC353577, purchased from BNCC) and *Segatella copri* (*S. copri*, BNCC354512, purchased from BNCC) are cultured in a strictly anaerobic environment at 37°C using Columbia Blood Plate medium (BNCC352241, BNCC).

### 4.9 Effect of Ionizing Radiation on *P. coprophilus*

For the study on the effect of ionizing radiation on *P. coprophilus*, the cultured *P. coprophilus* was subjected to ionizing radiation treatment (12 Gy). After 24 h, the supernatant of bacterial culture was collected for metabolomic analysis.

### 4.10 *S. copri* and *P. coprophilus* Intervention

Mice were intragastrically administered either live bacteria (2×10^8^ CFU/0.2 mL), inactivated bacteria (2×10^8^ CFU/0.2 mL), or PBS buffer. The intervention was conducted once daily for 7 consecutive days. Intestinal tissue samples were collected on day 7 and day 28 after the first administration for subsequent analysis.

### 4.11 *P. coprophilus* Supernatant Intervention

After the mice were exposed to ionizing radiation, they were intervened by intragastric administration of *P. coprophilus* supernatant (0.4 mL) twice a day for 28 consecutive days. Intestinal tissue samples from the mice were collected on days 7 and 28 post‐administration.

### 4.12 6‐Methyluracil Intervention

Mice received 6‐Methyluracil (50 mg kg^−1^) by gavage daily for 28 consecutive days. Intestinal tissue samples were collected on days 7 and 28 after the first administration for subsequent analysis.

### 4.13 Targeted Metabolomics

After thawing, 50 mg of intestinal tissue samples were weighed and extracted with 500 µL of methanol. 20 µL of internal standard mixed solution was added as the quantitative internal standard. The extract was vortexed for 3 min, refrigerated at ‐20°C for 30 min, then centrifuged at 12,000 r min^−1^ for 10 min at 4°C. 250 µL of the supernatant was collected, centrifuged again under the same conditions for 5 min, and 150 µL of the supernatant was transferred for further liquid chromatography‐mass spectrometry (LC‐MS) analysis. Tryptophan and its metabolites were analyzed using pre‐defined multiple reaction monitoring (MRM). Data collection was performed with Analyst 1.6.3 software (Sciex), and metabolite quantification was conducted using Multiquant 3.0.3 software (Sciex).

### 4.14 Untargeted Metabolomics Analysis

A 50 mg sample was weighed into a 2 mL grinding tube, followed by the addition of 0.5 mL of a methanol‐water solution (CH_3_OH:H_2_O v:v = 4:1), one stainless steel bead, and 200 µL of chloroform. The mixture was then ground twice in a ‐20°C pre‐cooled grinder (50 Hz, 3 min each), subjected to ultrasonic extraction for 30 min, and incubated at ‐20°C for 30 min. After low‐temperature centrifugation (13,000 rcf, 4°C, 15 min), the supernatant was transferred to a glass derivatization vial and dried under a nitrogen stream. For derivatization, 80 µL of methoxyamine hydrochloride in pyridine solution (15 mg mL^−1^) was added, vortex‐mixed for 2 min, and reacted at 37°C for 90 min for oximation. Subsequently, 80 µL of BSTFA (containing 1% TMCS) was added, vortex‐mixed for 2 min, and reacted at 70°C for 60 min. After cooling at room temperature for 30 min, non‐targeted metabolomic analysis was performed using a ThermoFisher Scientific TRACE 1610 GC‐Orbitrap Exploris high‐resolution gas chromatography‐mass spectrometry system (Shanghai Majorbio Bio‐pharm Technology Co., Ltd.).

### 4.15 Metagenomics

Fecal DNA was extracted from mouse feces, and the extracted genomic DNA was detected by 1% agarose gel electrophoresis. DNA was fragmented using a Covaris M220 and fragments of approximately 350 bp were screened. Library construction was performed using NEXTFLEX Rapid DNA‐Seq (Bioo Scientific, USA): “Y”‐shaped adapters were ligated; bead‐based screening was used to remove adapter self‐ligated fragments; PCR amplification was applied for library template enrichment; and PCR products were recovered by beads to obtain the final library. Sequencing was conducted on the DNBSEQ‐T7 platform using NBSEQ‐T7RS Reagent Kit (FCL PE150) version 3.0.

### 4.16 Isolation and Culture of Mouse Small Intestinal Primary Fibroblasts

After anesthetizing the mice, the small intestine was removed from the abdominal cavity and placed in a petri dish. The mesentery was carefully dissected, and the intestinal lumen was rinsed with pre‐cooled DPBS containing double antibiotics. The intestine was then longitudinally incised, the mucus inside was scraped off, and the lumen was repeatedly rinsed with pre‐cooled DPBS. The intestine was cut into fragments (approximately 2–5 mm), added to 20 mL of pre‐warmed extraction solution (a mixture of DPBS, FBS, EDTA, and DTT), and vigorously shaken at 37°C for 15 min. The tissue fragments were transferred to digestion solution containing collagenase D and pronase, followed by shaking at 37°C for 30 min. After digestion, the tissue was ground and filtered through a 70‐µm cell strainer. The filtrate was centrifuged to discard the supernatant, and the cells were resuspended in lysis buffer (a mixture of ddH2O, NH4Cl, KHCO3, and EDTA). Following lysis and another centrifugation to remove the supernatant, the cells were resuspended in complete medium, filtered again through a cell strainer, and transferred to culture flasks for cultivation.

### 4.17 Cell Kyn Intervention

Cells were exposed to different concentrations of Kyn (0, 25, 50, 100 µm), subjected to ionizing radiation treatment (12 Gy), and then RNA and proteins were extracted from the cells 24 h later.

### 4.18 RNAi Knockdown

According to the manufacturer's protocol (D‐Nano Therapeutics, Beijing, China), the fibroblasts were transfected with 50 nm Ahr siRNA and then used for subsequent experiments. The sequences of siRNA were shown in Table S3.

### 4.19 ELISA

The concentration of kynurenine (Kyn) was determined using a commercial KYN ELISA Kit (EU0188, FineTest). For intestinal tissue, samples were homogenized in ice‐cold PBS and centrifuged at 12,000 × g for 10 min at 4°C. For fibroblast culture medium, the conditioned medium was collected and centrifuged at 3,000 × g for 10 min at 4°C. The Kyn concentration in the resulting supernatants was measured according to the kit instructions.

### 4.20 IEC6 Cell EMT Assay

IEC6 were cultured in DMEM with 5% FBS. Cells were seeded in 6‑well plates. Four groups were established: Control, Kyn (100 µM), IR (12 Gy), and IR + Kyn. After 48 h, cells were harvested for total protein extraction. Expression of epithelial marker E‐cadherin and mesenchymal marker Vimentin was analyzed by Western blot.

### 4.21 Real‐Time Quantitative RT‐PCR

RNA was extracted from cell or tissue samples using the RNA extraction kit of Foregene Co., Ltd. cDNA synthesis was then completed with the reverse transcription kit of Yeasen Biotechnology. Subsequently, real‐time fluorescent quantitative PCR was performed using the company's qPCR SYBR Green Master Mix, and experimental data were analyzed by Agilent AriaMx software. The primers sequences are detailed in Table S4.

### 4.22 Western Blot

Cell samples were directly lysed by adding RIPA lysis buffer, whereas intestinal tissue samples underwent homogenization after the addition of RIPA lysis buffer to ensure thorough lysis. The protein concentration was measured using a BCA protein quantification kit and adjusted to a uniform level. Following denaturation at 95°C, the protein samples were separated by SDS‐PAGE gel electrophoresis using a 7.5%‐12% concentration gradient gel, and then transferred to a PVDF membrane. After blocking treatment of the PVDF membrane, it was sequentially incubated with primary and secondary antibodies, developed via ECL chemiluminescence, and finally, the band intensities were quantitatively analyzed using ImageJ software. The antibodies used in western blot were shown in Table S5.

### 4.23 Histology and Immunohistochemistry

Intestinal tissue samples were fixed with tissue fixative for 24 h, followed by paraffin embedding and preparation of 4 µm continuous sections. The sections were subjected to Masson's trichrome staining and Collagen III immunohistochemical staining, respectively. Image quantitative analysis was performed using ImageJ software.

### 4.24 Immunofluorescence

Paraffin‐embedded intestinal sections (4 µm) were deparaffinized, rehydrated, and subjected to antigen retrieval. After blocking, sections were incubated overnight at 4°C with primary antibodies against IDO1 in combination with antibodies against CD11c, EPCAM, or CD45. After washing, sections were incubated with appropriate Alexa Fluor‐conjugated secondary antibodies for 1 h at room temperature. Nuclei were counterstained with DAPI.

### 4.25 Immunofluorescence (for Cells)

Primary intestinal fibroblasts were treated as indicated. Cells were fixed with 4% paraformaldehyde for 15 min and permeabilized with 0.1% Triton X‐100 for 10 min. After blocking with 5% BSA for 1 h, samples were incubated overnight at 4°C with anti‐AHR antibody. After washing, samples were incubated with Alexa Fluor 488‐conjugated secondary antibody for 1 h at room temperature in the dark. Nuclei were counterstained with DAPI. Images were captured using a confocal microscope.

### 4.26 Co‐Immunoprecipitation (Co‐IP) and Ubiquitination Assay

Primary intestinal fibroblasts were treated as indicated. To capture ubiquitinated proteins, cells were treated with 10 µm MG132 (MCE) for 6 h prior to harvest. Cells were lysed in IP lysis buffer (Beyotime). Protein concentrations were determined by BCA assay. For each IP, 500 µg of total protein lysate was incubated with 2 µg of anti‐AHR antibody overnight at 4°C with gentle rotation. Protein A/G magnetic beads (Beyotime) were then added and incubated for 2 h. Beads were washed four times with lysis buffer, and bound proteins were eluted by boiling loading buffer. The eluates were then subjected to western blot analysis using anti‐Ubiquitin and anti‐AHR antibodies.

### 4.27 Statistical Analysis

Data were analyzed using GraphPad Prism 10.4 software and are presented as mean ± standard deviation (SD). Intergroup differences were assessed using Student's t‐test or one‐way analysis of variance (ANOVA) for normally distributed data, and the Mann‐Whitney U test for non‐normally distributed data. Statistical significance was defined as *p* < 0.05 (ns = non‐significant; * < 0.05).

## Conflicts of Interest

The authors declare no conflicts of interest.

## Supporting information




**Supporting File**: advs73966‐sup‐0001‐SuppMat.docx.

## Data Availability

The data that support the findings of this study are available from the corresponding author upon reasonable request.
