# Supplementary material for: Phocaeicola coprophilus‐Derived 6‐Methyluracil Attenuates Radiation‐Induced Intestinal Fibrosis by Suppressing the IDO1‐Kynurenine‐AHR Axis
Source: Adv Sci (Weinh). 2026 Jan 20;13(18):e18502. doi: 10.1002/advs.202518502 (PMC13042846; doi:10.1002/advs.202518502)
Supplement: Supplementary file 1 — Supporting File: advs73966‐sup‐0001‐SuppMat.docx. [file ADVS-13-e18502-s001.docx]

***Phocaeicola coprophilus*-Derived 6-Methyluracil Attenuates Radiation-Induced Intestinal Fibrosis by Suppressing the IDO1-Kynurenine-AHR Axis**

Jiaxin Zhang, Zhen Wang, Shuang Li, Chao Luo, Han Li, Shengjie Ma, Pai Wang, Heshi Liu, Lijun Sun, Yue Yin, Weizhen Zhang^*^, Quan Wang^*^.

J. Zhang, Z. Wang, S. Li, S. Ma, P. Wang, H. Liu, Q. Wang

Department of Gastrocolorectal Surgery, General Surgery Center, The First Hospital of Jilin University, Changchun 130021, China.

E-mail: wquan@jlu.edu.cn

C. Luo, H. Li, L. Sun, W. Zhang

Department of Physiology and Pathophysiology, School of Basic Medical Sciences, and State Key Laboratory of Vascular Homeostasis and Remodeling, Peking University, Beijing, 100191, People's Republic of China.

E-mail: weizhenzhang@bjmu.edu.cn

Y. Yin

Department of Pharmacology, School of Basic Medical Sciences, and State Key Laboratory of Vascular Homeostasis and Remodeling, Peking University, Beijing, 100191, People's Republic of China.

**Supplemental Figures**


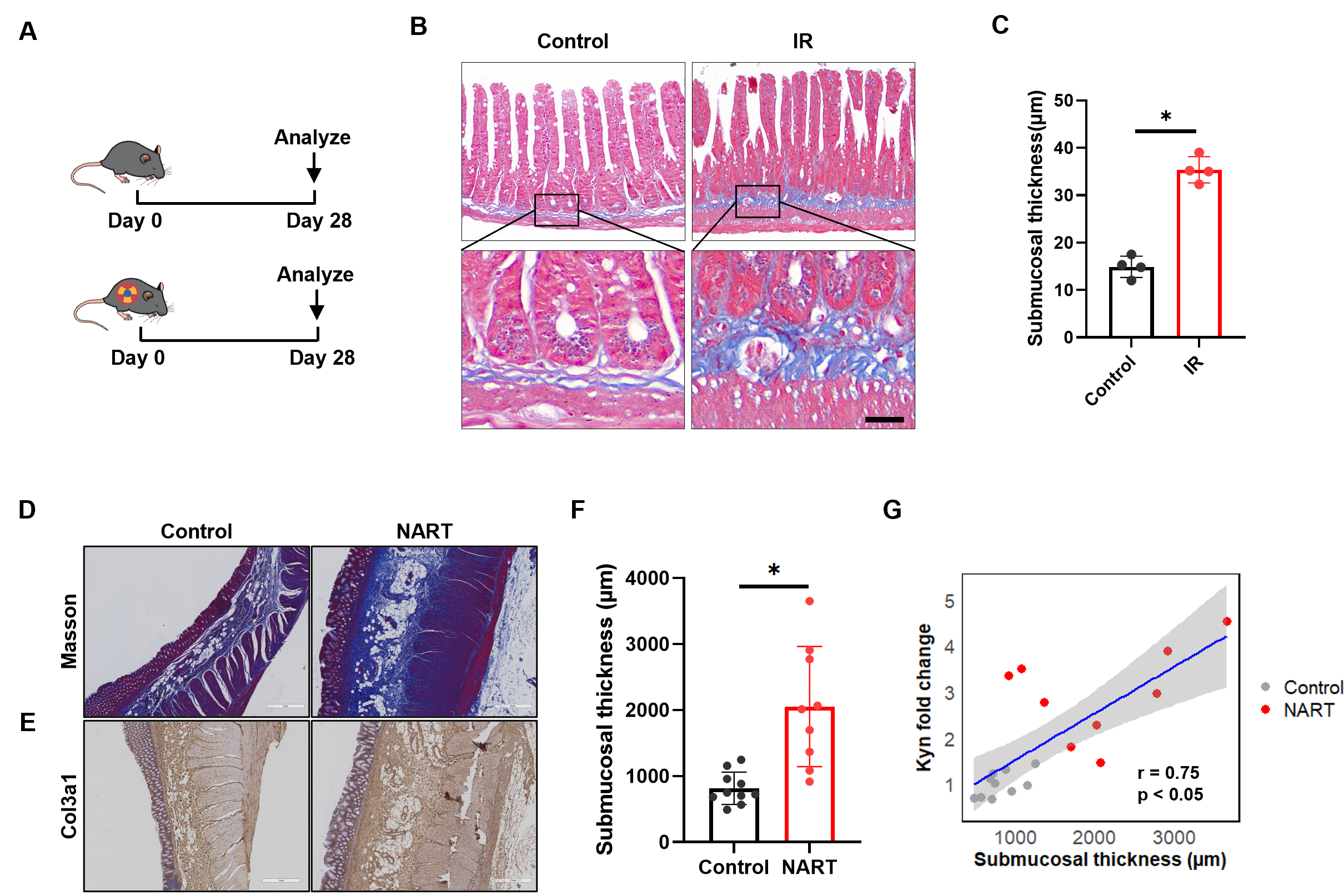


**Figure S1**. Radiation-induced intestinal fibrosis (RIF) in mouse and human samples. A) Mice received 12 Gy abdominal IR. Intestinal tissue samples were harvested from the mice on days 28 after the IR (n=6). B) Representative Masson's trichrome staining of small intestinal tissue (scale bar = 20 μm). C) Quantitative analysis of intestinal submucosal thickness (n=4). D) Representative Masson's trichrome staining of clinical intestinal tissues from non-radiotherapy controls and radiotherapy patients. E) Representative COL3A1 staining of clinical intestinal tissues from non-radiotherapy controls and radiotherapy patients. F) Quantitative analysis of submucosal thickness in human intestinal tissue (n=9-10). G) Spearman correlation analysis for Kyn levels and the submucosal thickness in human intestinal tissue (n=9–10). Data are presented as mean ± SD. *p < 0.05.


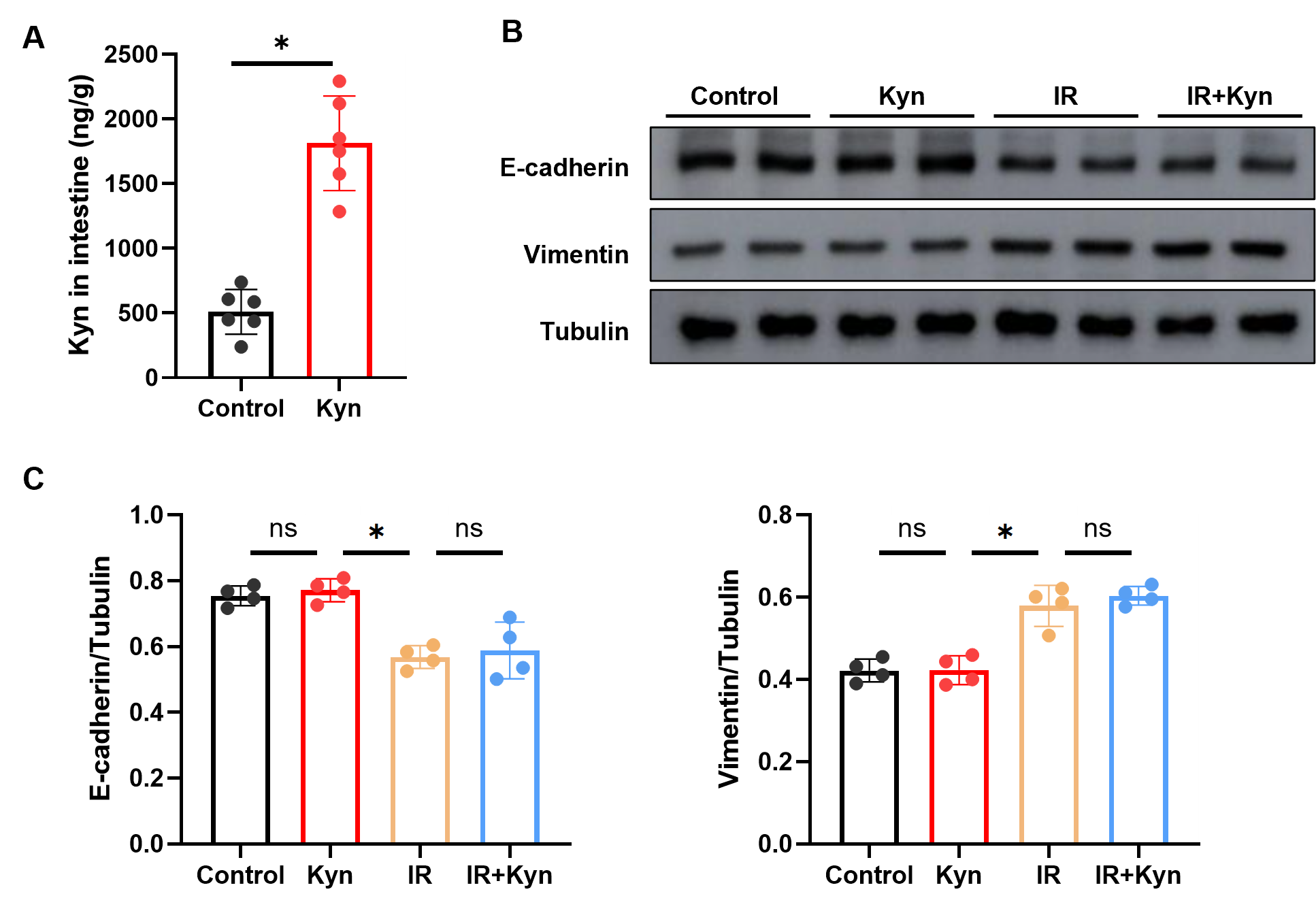


**Figure S2**. Pharmacokinetics of exogenous Kyn and its effect on epithelial-mesenchymal transition (EMT). A) Intestinal Kyn concentration 6 hours after a single intraperitoneal injection of Kyn (50 mg/kg) in naïve mice (n=6). B, C) Western blot analysis of EMT markers in IEC6 cells treated with Kyn and/or IR (n=4). Data are presented as mean ± SD. *p < 0.05.


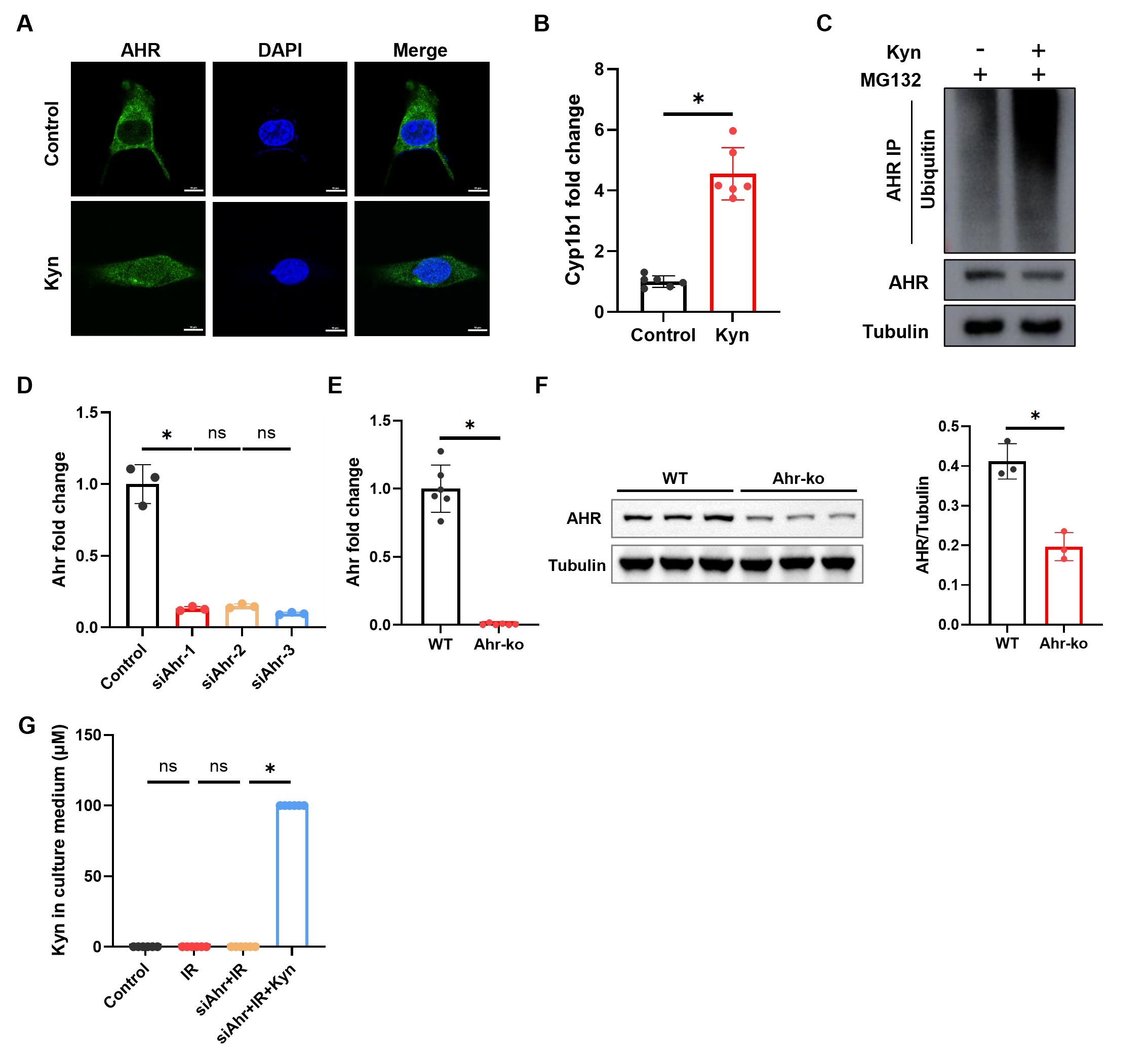


**Figure S3**. AHR activation and validation of Ahr knockdown and knockout models. A) Immunofluorescence staining of AHR (green) and DAPI (blue) in primary intestinal fibroblasts treated with Kyn (scale bar = 10 μm). B) qRT-PCR analysis of Cyp1b1 mRNA expression in fibroblasts after Kyn treatment (n=6). C) Co-immunoprecipitation assay showing poly-ubiquitination of AHR in fibroblasts treated with Kyn. D) Validation of Ahr gene knockdown efficiency by three independent siRNAs via qRT-PCR (n=3). E) qRT-PCR analysis of Ahr mRNA expression in control and Ahr-ko intestinal tissue (n=6). F) Western blot analysis of total AHR protein expression in control and Ahr-ko intestinal tissue (n=3). G) Kyn concentration in fibroblast culture medium under indicated treatments (n=6). Data are presented as mean ± SD. *p < 0.05.


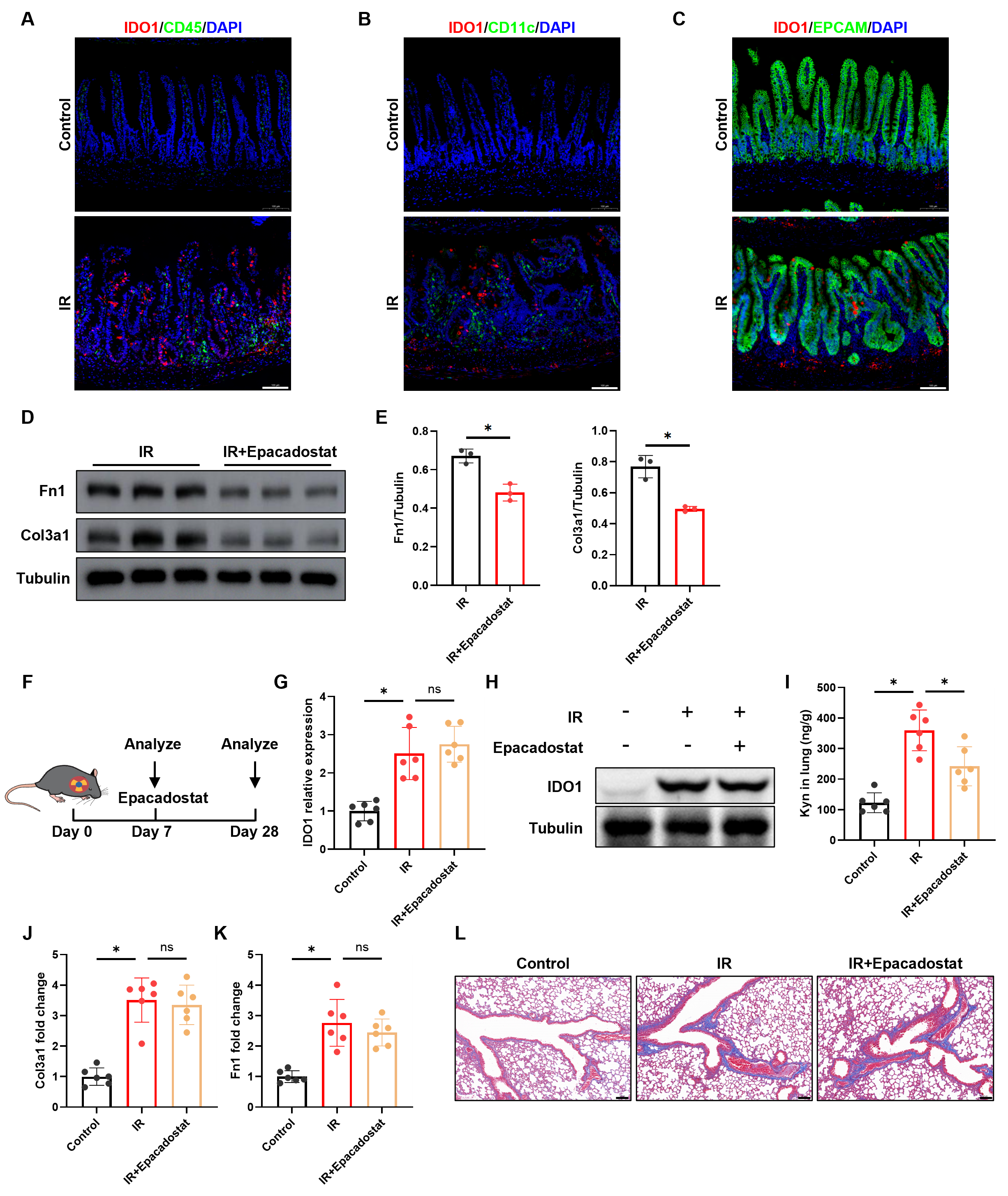


**Figure S4**. Inhibition of IDO1 attenuates RIF. A) Representative IDO1/CD45/DAPI immunostaining images of intestine at day 7 after IR (scale bar = 100 μm). B) Representative IDO1/CD11c/DAPI immunostaining images of intestine at day 7 after IR (scale bar = 100 μm). C) Representative IDO1/EPCAM/DAPI immunostaining images of intestine at day 7 after IR (scale bar = 100 μm). D, E) Western blot analysis of FN1 and COL3A1 in intestinal tissues from mice treated with Epacadostat (n=3). F) Experimental scheme for thoracic irradiation-induced pulmonary fibrosis model with Epacadostat treatment (n=6) (G-L). G) qRT-PCR analysis of IDO1 mRNA in lung tissue at day 7 post-IR (n=6). H) Western blot analysis of IDO1 levels in lung tissue at day 7 post-IR. I) ELISA measurement of Kyn levels in lung tissue at day 7 post-IR (n=6). J) qRT-PCR analysis of Col3a1 mRNA in lung tissue at day 28 post-IR (n=6). K) qRT-PCR analysis of Fn1 mRNA in lung tissue at day 28 post-IR (n=6). L) Representative Masson’s trichrome staining of lung tissue (scale bar = 100 μm). Data are presented as mean ± SD. *p < 0.05.


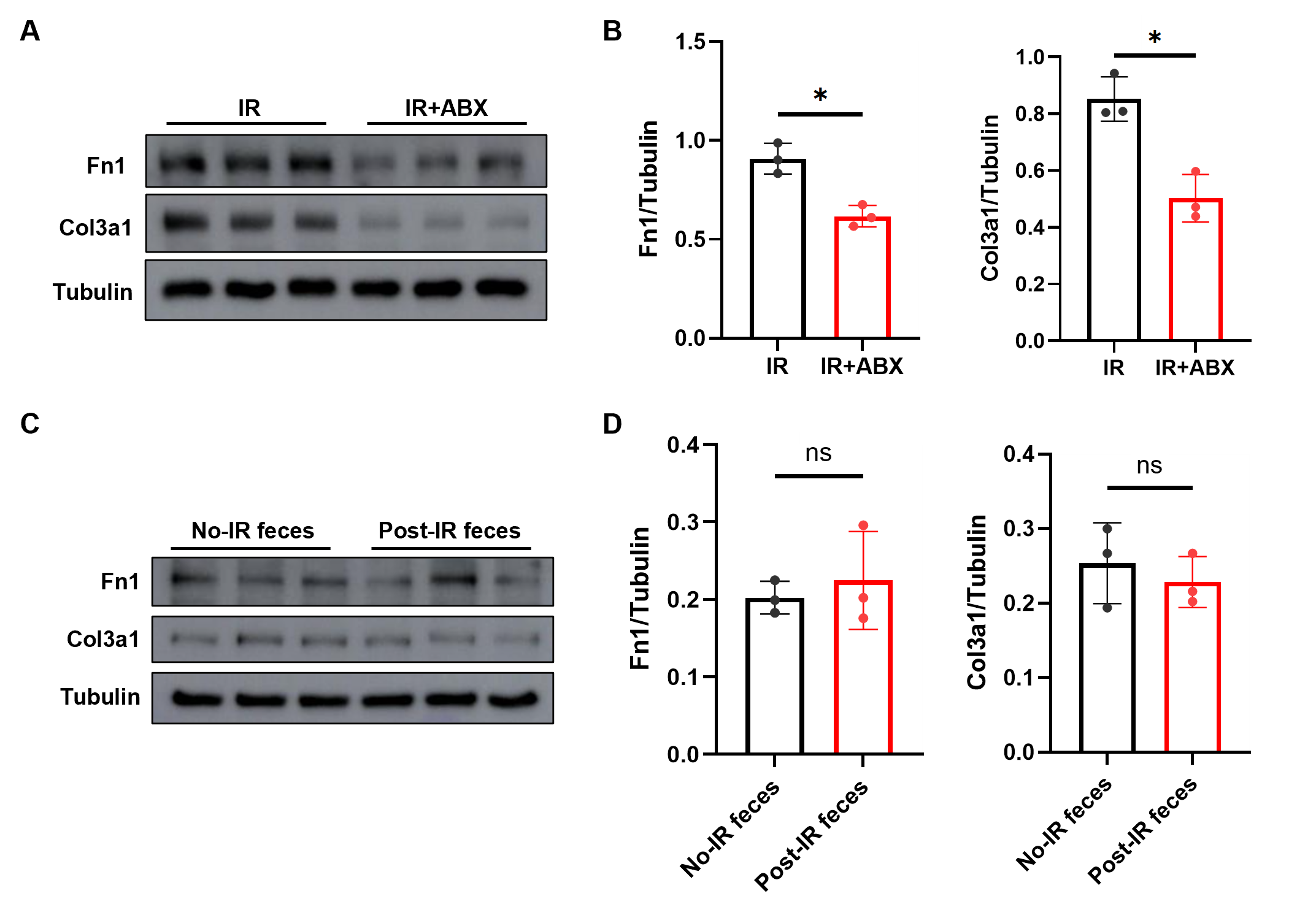


**Figure S5**. Gut microbiota depletion and FMT effects on fibrosis. A, B) Western blot analysis of FN1 and COL3A1 in intestinal tissue from antibiotic-treated mice (n=3). C, D) Western blot analysis of FN1 and COL3A1 in intestinal tissue from FMT recipient mice (n=3). Data are presented as mean ± SD. *p < 0.05.


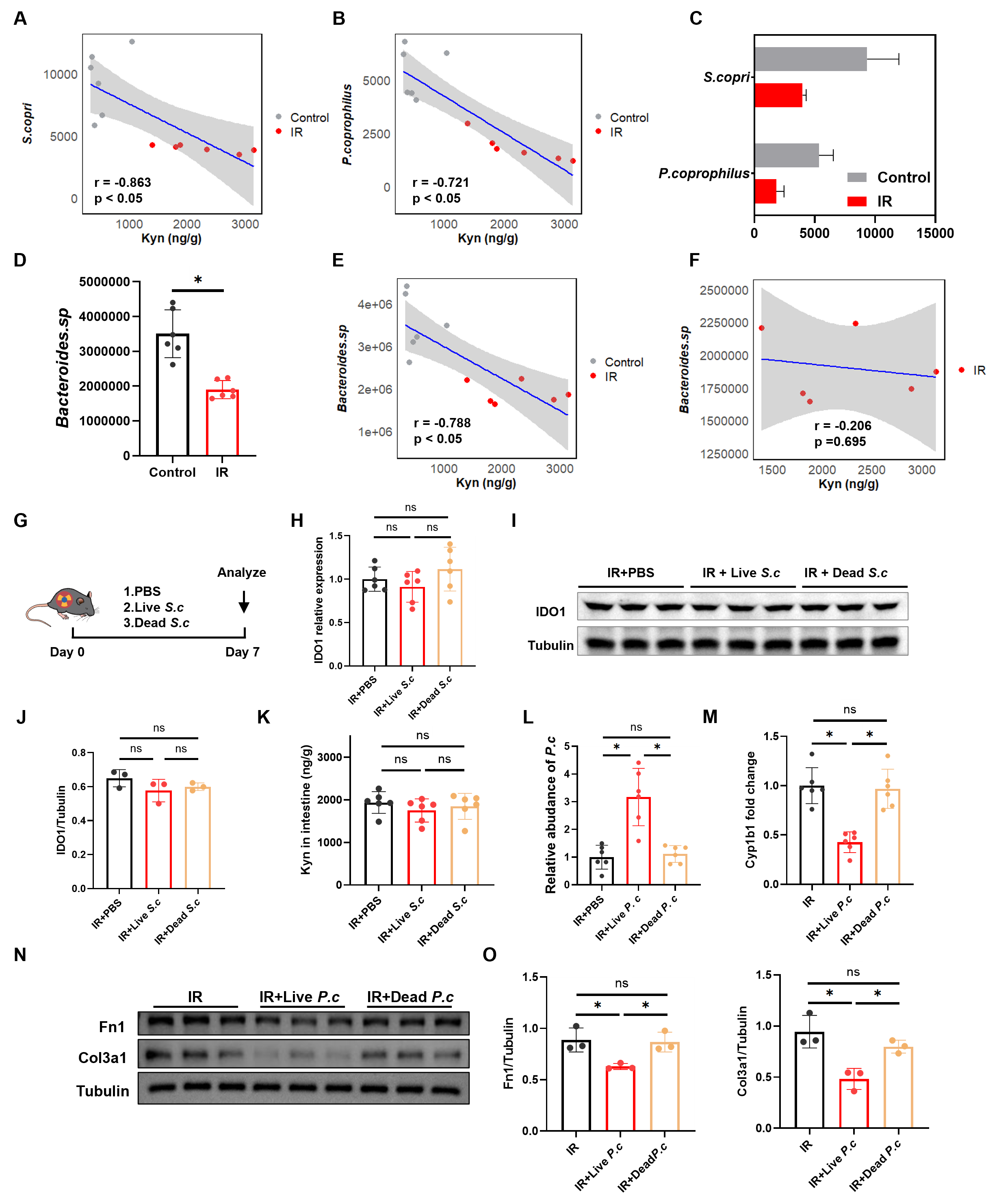


**Figure S6**. A) Spearman correlation analysis between fecal S. copri abundance and intestinal tissue Kyn concentration in the whole cohort (n=6). B) Spearman correlation analysis between fecal P. coprophilus abundance and intestinal tissue Kyn concentration in the whole cohort (n=6). C) The two most abundant bacterial species that show significant negative correlation with Kyn levels in the intestine based on Spearman correlation analysis. Metagenomic analysis and validation of candidate bacterial species. D) Relative abundance of *Bacteroides.sp* in feces (n=6). E) Spearman correlation analysis between fecal *Bacteroides.sp* abundance and intestinal tissue Kyn concentration in the whole cohort (n=6). F) Spearman correlation analysis between fecal *Bacteroides.sp* abundance and intestinal tissue Kyn concentration in the IR group (n=6). G) Experimental scheme for (H)-(K). Intervention with live or inactivated *S. copri* (n=6). H) qRT-PCR analysis of IDO1 mRNA levels in intestinal tissues (n=6). I, J) Western blot analysis of IDO1 protein levels in intestinal tissues (n=3). K) ELISA detection of Kyn levels in intestinal tissues. L) Quantitation of *P. coprophilus* in mouse feces by qRT-PCR at day 7 after IR (n=6). M) qRT-PCR of Cyp1b1 mRNA in primary intestinal fibroblasts from *P. coprophilus* supplemented mice at day 7 after IR (n=6). N, O) Western blot analysis of FN1 and COL3A1 in intestinal tissue from *P. coprophilus* supplemented mice (n=3). Data are presented as mean ± SD. *p < 0.05.


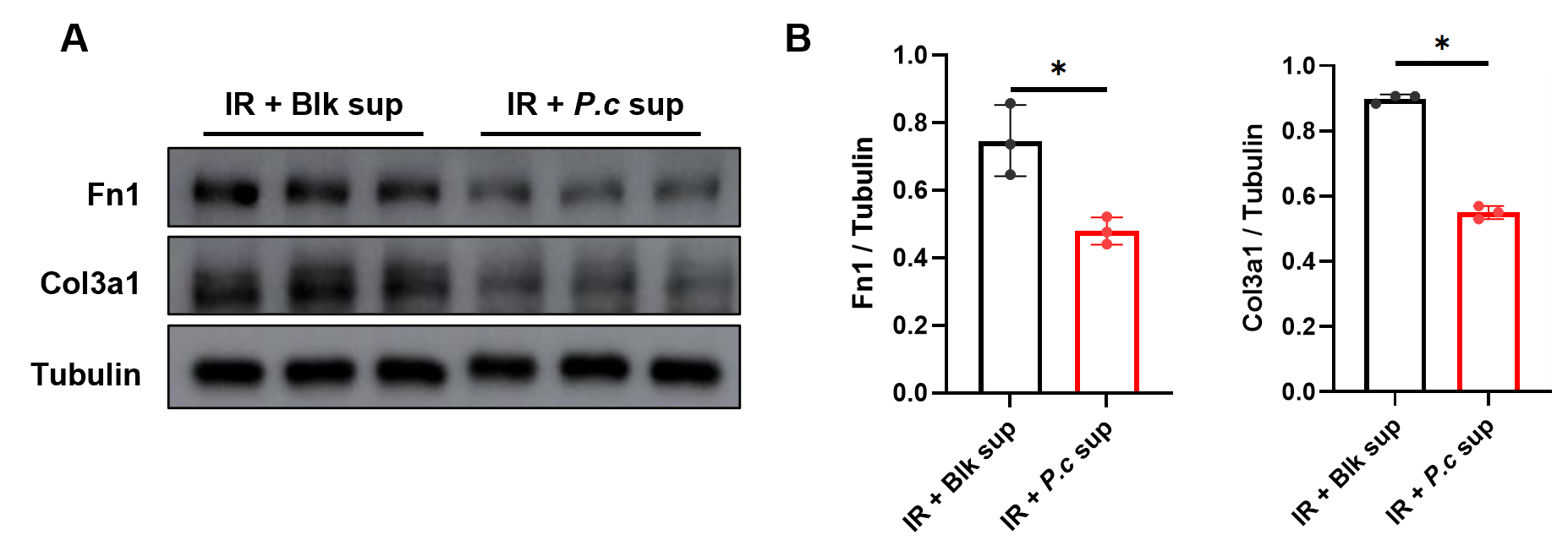


**Figure S7.** Effects of *P. coprophilus* supernatant on fibrosis.

A, B) Western blot analysis of FN1 and COL3A1 in intestinal tissue from mice treated with *P. coprophilus* supernatant (P. c sup) (n=3). Data are presented as mean ± SD. *p < 0.05.

**
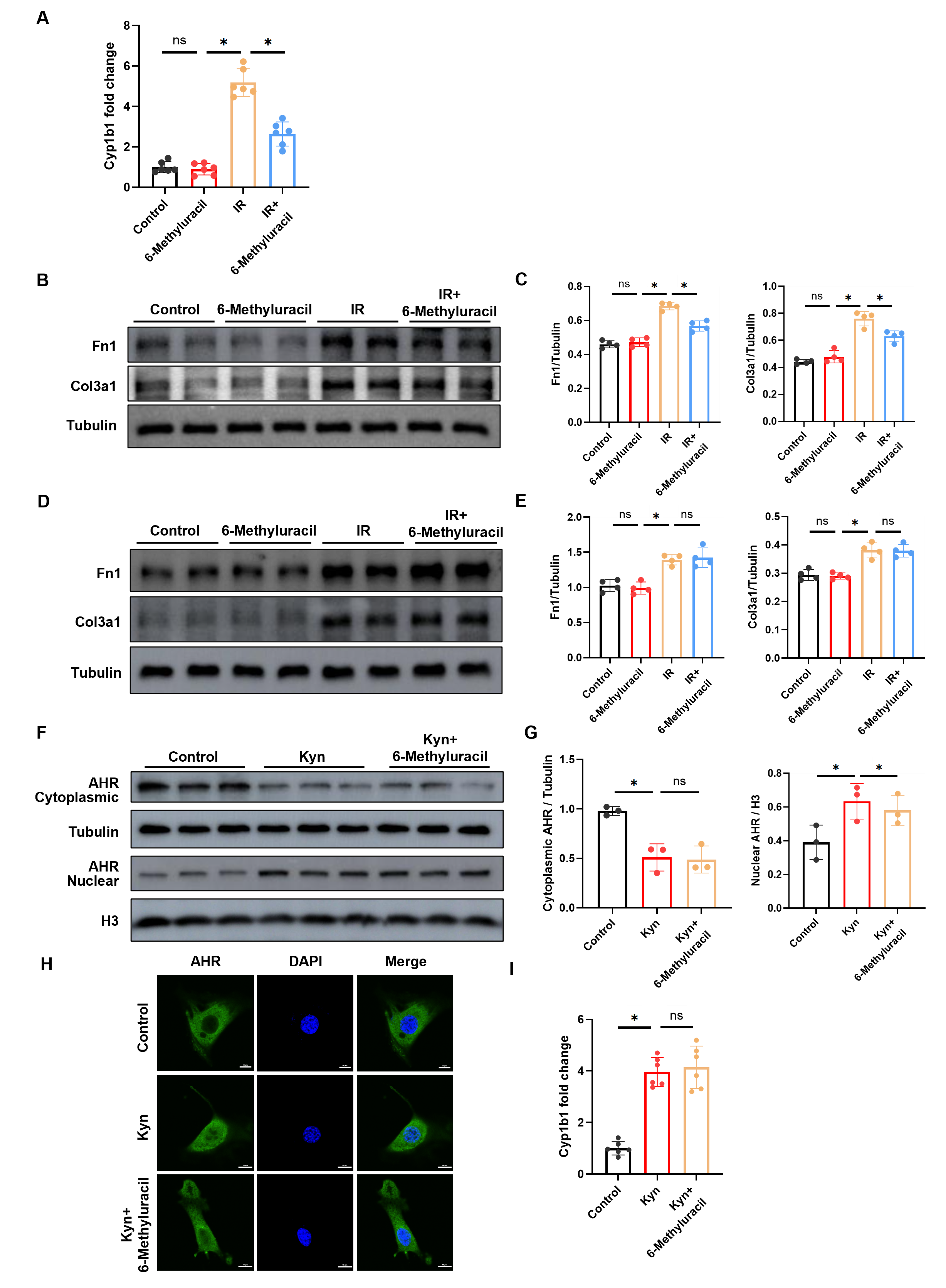
**

**Figure S8.** In vitro and in vivo effects of 6-Methyluracil on fibroblast activation and AHR signaling. A) qRT-PCR of Cyp1b1 mRNA in primary intestinal fibroblasts from 6-Methyluracil treated mice (n=6). B, C) Western blot analysis of FN1 and COL3A1 in intestinal tissue from 6-Methyluracil treated mice (n=4). D-I) Effects of 6-Methyluracil on primary intestinal fibroblasts in vitro. D, E) Western blot of FN1 and COL3A1 protein (n=4). F, G) Western blot analysis of total AHR protein (n=3). H) Immunofluorescence of AHR (green) and DAPI (blue) (scale bar = 10 μm). I) qRT-PCR of Cyp1b1 mRNA (n=6). Data are presented as mean ± SD. *p < 0.05.


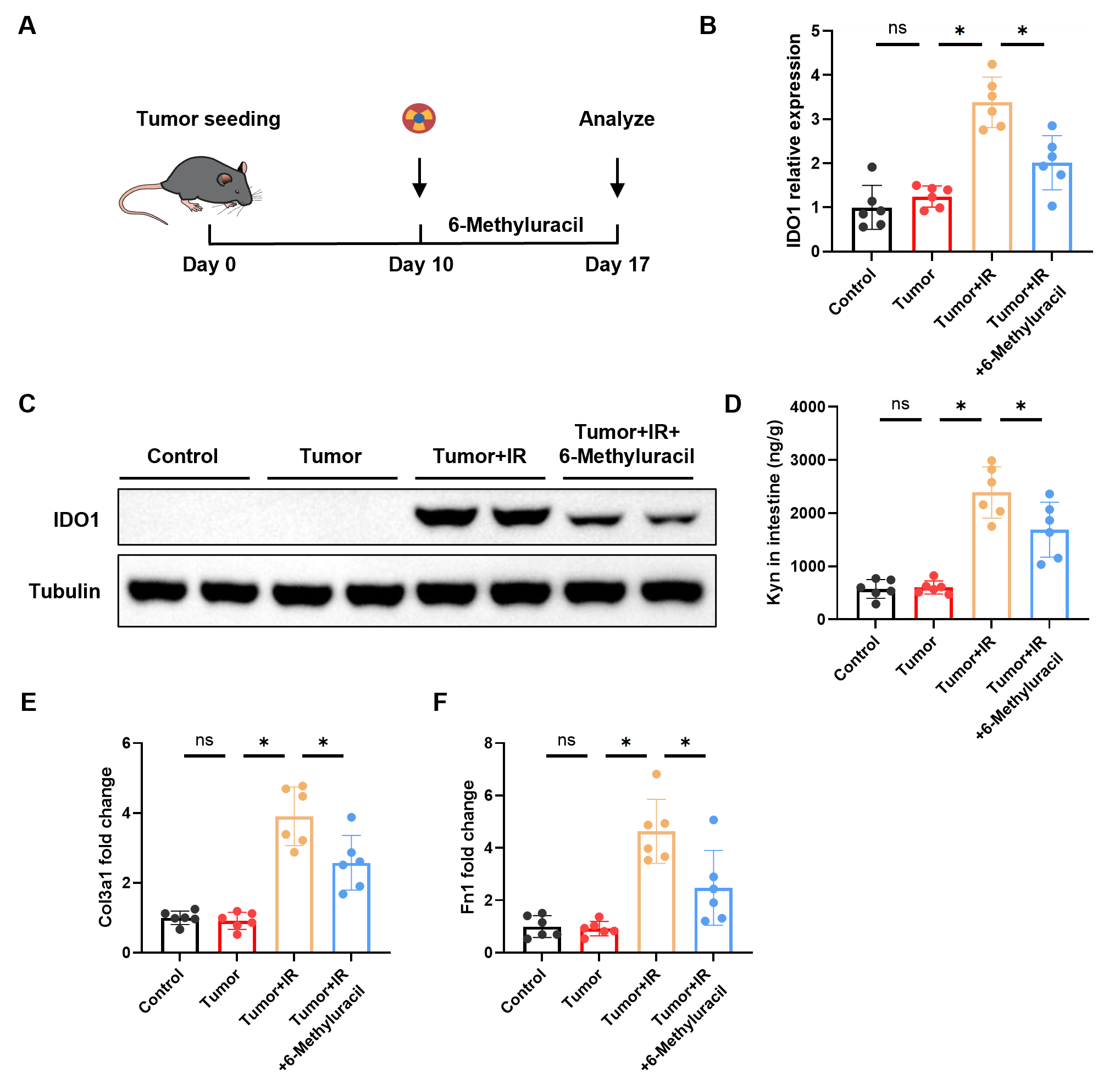


**Figure S9.** 6-Methyluracil suppresses radiation-induced activation of the IDO1-Kyn axis in the intestine of tumor-bearing mice. A) Experimental timeline: MC38 colon cancer-bearing mice received abdominal irradiation (12 Gy) with or without 6-Methyluracil treatment. All molecular analyses were performed on non-tumorous intestinal tissue. B) qRT-PCR analysis of IDO1 mRNA in intestinal tissue (n=6). C) Western blot analysis of IDO1 protein in intestinal tissue. D) ELISA measurement of Kyn concentration in intestinal tissue (n=6). E, F) qRT-PCR analysis of fibrosis-related genes Col3a1 and Fn1 in intestinal tissue (n=6).

**Supplemental Tables**

**Table S1. Results of LC/MS targeting metabolites in mouse intestine following IR**

| Compounds | Tryptophan  （ng/g） | Kynurenine  （ng/g） | Xanthurenic acid  （ng/g） | Quinolinic acid  （ng/g） |
| --- | --- | --- | --- | --- |
| Day0-1 | 26781.9212 | 393.485481 | 100.47136 | 137.617343 |
| Day0-2 | 19319.3567 | 534.874593 | 65.0372391 | 146.423511 |
| Day0-3 | 18256.4809 | 1041.44723 | 111.312513 | 207.758726 |
| Day0-4 | 24877.71 | 465.876658 | 96.7887813 | 191.84277 |
| Day0-5 | 35650.6692 | 351.417817 | 94.1344626 | 114.256587 |
| Day0-6 | 19108.7987 | 327.396456 | 125.628477 | 127.489182 |
| Day3-1 | 26307.7444 | 439.013553 | 142.736689 | 89.709971 |
| Day3-2 | 23445.2266 | 939.318226 | 85.7799421 | 106.842816 |
| Day3-3 | 17003.678 | 925.644667 | 73.9249081 | 109.110135 |
| Day3-4 | 14807.2266 | 983.779297 | 56.9177734 | 131.617188 |
| Day3-5 | 9572.95772 | 2138.04392 | 45.403633 | 115.01642 |
| Day3-6 | 11830.4505 | 1016.90467 | 88.741251 | 101.300282 |
| Day7-1 | 21597.5413 | 3141.60621 | 60.2814389 | 179.818622 |
| Day7-2 | 18577.4445 | 2332.33361 | 92.4568611 | 172.955012 |
| Day7-3 | 19077.9835 | 1875.47325 | 55.8845679 | 116.618313 |
| Day7-4 | 17712.1331 | 2893.50995 | 62.9748867 | 200.6234 |
| Day7-5 | 31129.5649 | 1801.45688 | 176.536519 | 219.996115 |
| Day7-6 | 31332.7735 | 1392.77766 | 88.804535 | 229.892925 |
| Day14-1 | 26445.8732 | 2292.55383 | 79.569378 | 199.119817 |
| Day14-2 | 25927.502 | 1224.27108 | 58.7611308 | 176.761229 |
| Day14-3 | 25652.2634 | 1778.26132 | 76.6463992 | 268.641975 |
| Day14-4 | 26211.6349 | 1805.87763 | 73.1975928 | 240.707121 |
| Day14-5 | 21303.7813 | 2141.11481 | 60.989518 | 240.817006 |
| Day14-6 | 18475.1178 | 1563.42412 | 64.0903133 | 173.403645 |
| Day28-1 | 25045.4265 | 1016.24124 | 72.2593737 | 268.447672 |
| Day28-2 | 21866.3757 | 684.14069 | 62.6878637 | 208.648171 |
| Day28-3 | 25158.5293 | 545.520128 | 84.9287565 | 155.069749 |
| Day28-4 | 21776.3183 | 1206.11697 | 59.1222435 | 209.377756 |
| Day28-5 | 23439.7 | 513.658 | 72.4471 | 143.325 |
| Day28-6 | 18859.2108 | 1164.35653 | 57.4421971 | 217.103906 |

**Table S2. Clinical characteristics of the patients with rectal cancer**

| Characteristics | No-NART  (n=10) | NART  (n=9) |
| --- | --- | --- |
| Gender, n (%) |  |  |
| Male | 6 (60%) | 7(77.7%) |
| Female | 4 (40%) | 2(22.2%) |
| Age (y, mean ± SD) | 56.8±8.94 | 52.22±7.68 |
| CRP (mg/L, mean ± SD) | 3.04±0.49 | 4.28±1.22 |
| Hemoglobin (g/L, mean ± SD) | 136.40±22.81 | 135.67±34.25 |
| Albumin (g/L, mean ± SD) | 41.19±5.11 | 38.34±4.38 |
| Use of concomitant chemotherapy n (%) | 0 (0%) | 9 (100%) |
| Time between the completion of radiotherapy and surgery (week, mean ± SD) | 0 | 6.78±1.47 |

**Table S3. Primer sequences used in RNAi Knockdown**

| siRNA | Forward Primer (5'->3') | Reverse Primer (5'->3') |
| --- | --- | --- |
| siAhr-1 | GACAUCGACAUAACGGACGAA | CGUCCGUUAUGUCGAUGUCUU |
| siAhr-2 | GACCAGAUUACAUCAUCGCCA | GCGAUGAUGUAAUCUGGUCUU |
| siAhr-3 | GUAUCUUCAUGGACAGAACAA | GUUCUGUCCAUGAAGAUACUU |

**Table S4. Primer sequences used in qRT-PCR**

| Gene | Forward Primer (5'->3') | Reverse Primer (5'->3') |
| --- | --- | --- |
| M-Ido1 | AGAGGACACAGGTTACAGCG | CATCGTCATCCCCTCGGTTC |
| M-Col3a1 | ACGTAAGCACTGGTGGACAG | CAGGAGGGCCATAGCTGAAC |
| M-Fn1 | GAGTGGAAGTGTGAGCGACA | GGTGAGTCTGCGGTTGGTAA |
| M-Ahr | CTGAGGGCCAAGAGCTTCTTTG | CTGCCAGTCTCTGATTTGTGCTC |
| M-Cyp1b1 | CAACTCCGTGAGAAAGCCCT | GCTTCAACCTGAGAGTCCCC |
| M-Actin | GTGACGTTGACATCCGTAAAGA | GCCGGACTCATCGTACTCC |
| H-IDO1 | GCAGCGTCTTTCAGTGCTTT | ACAAACTCACGGACTGAGGG |
| H-COL3A1 | CGCCCTCCTAATGGTCAAGG | TTCTGAGGACCAGTAGGGCA |
| H-FN1 | TGCACAACCAATGAAGGGGT | GTCCATTCCCCACGACCATT |
| H-ACTB | CTCGCCTTTGCCGATCC | ATCCTTCTGACCCATGCCC |
| 16s | GTGSTGCAYGGYTGTCGTCA | ACGTCRTCCMCACCTTCCTC |
| *P. coprophilus* | CGTATCCAACCTTCCGCTTACTCG | ACTGCCTAATGGAACGCATC |

**Table S5. Antibodies used in Western Blot, Immunohistochemistry, and Immunofluorescence**

| Antibody | Source | Catalog |
| --- | --- | --- |
| IDO1 | Proteintech | Cat No.13268-1-AP |
| COL3A1 | Proteintech | Cat No.22734-1-AP |
| FN1 | Proteintech | Cat No.15613-1-AP |
| AHR | Proteintech | Cat No.67785-1-lg |
| TUBULIN | Proteintech | Cat No.11224-1-AP |
| H3 | Proteintech | Cat No.17168-1-AP |
| E-cadherin | Servicebio | Cat No.GB11082-50 |
| Vimentin | Servicebio | Cat No.GB11192-50 |
| Ubiquitin | HUABIO | Cat No.ET1610-39 |
| CD11c | Servicebio | Cat No.GB12059-50 |
| EPCAM | Servicebio | Cat No.GB12274-50 |
| CD45 | Proteintech | Cat No.60287-1-Ig |
